# Machine Learning and Computed Tomography Radiomics to Predict Disease Progression to Upfront Pembrolizumab Monotherapy in Advanced Non-Small-Cell Lung Cancer: A Pilot Study

**DOI:** 10.3390/cancers17010058

**Published:** 2024-12-28

**Authors:** Ian Janzen, Cheryl Ho, Barbara Melosky, Qian Ye, Jessica Li, Gang Wang, Stephen Lam, Calum MacAulay, Ren Yuan

**Affiliations:** 1 Integrative Oncology, BC Cancer Research Institute, 675 West 10th Avenue, Vancouver, BC V5Z Il3, Canada; ijanzen@bccrc.ca (I.J.);; 2 Interdisciplinary Oncology Program, Faculty of Medicine, University of British Columbia, 2329 West Mall, Vancouver, BC V6T IZ4, Canada; 3 BC Cancer, Vancouver Center, 600 West 10th Avenue, Vancouver, BC V5Z 4E6, Canada; 4 Department of Medical Oncology, Faculty of Medicine, University of British Columbia, 2329 West Mall, Vancouver, BC V6T IZ4, Canada; 5 Department of Statistics, Faculty of Science, University of British Columbia, 2329 West Mall, Vancouver, BC V6T 1Z4, Canada; 6 Department of Radiology, Faculty of Medicine, University of British Columbia, 2329 West Mall, Vancouver, BC V6T IZ4, Canada; 7 Department of Pathology, Faculty of Medicine, University of British Columbia, 2329 West Mall, Vancouver, BC V6T IZ4, Canada; 8 Department of Respirology, Faculty of Medicine, University of British Columbia, 2329 West Mall, Vancouver, BC V6T IZ4, Canada

**Keywords:** non-small- cell lung cancer (NSCLC), immunotherapy, treatment response, peritumoral, radiomics

## Abstract

Background/Objectives: Pembrolizumab monotherapy is approved in Canada for first-line treatment of advanced NSCLC with PD-L1 ≥ 50% and no EGFR/ALK aberrations. However, approximately 55% of these patients do not respond to pembrolizumab, underscoring the need for the early intervention of non-responders to optimize treatment strategies. Distinguishing the 55% sub-cohort prior to treatment is a real-world dilemma. Methods: In this retrospective study, we analyzed two patient cohorts treated with pembrolizumab monotherapy (training set: *n* = 97; test set: *n* = 17). The treatment response was assessed using baseline and follow-up CT scans via RECIST 1.1 criteria. Results: A logistic regression model, incorporating pre-treatment CT radiomic features of lung tumors and clinical variables, achieved high predictive accuracy (AUC: 0.85 in training; 0.81 in testing, 95% CI: 0.63–0.99). Notably, radiomic features from the peritumoral region were found to be independent predictors, complementing the standard CT evaluations and other clinical characteristics. Conclusions: This pragmatic model offers a valuable tool to guide first-line treatment decisions in NSCLC patients with high PD-L1 expression and has the potential to advance personalized oncology and improve timely disease management.

## Simple Summary

Lung cancer is Canada’s leading cause of cancer deaths, with most cases diagnosed at advanced stages where treatment hinges on target mutations. While upfront immunotherapy single pembrolizumab is standard for high PD-L1 lung cancers, nearly 50% of patients do not respond, risking disease progression and worse survival. Therefore, identifying these patients for alternative regimens is critical. To address this, we developed a predictive risk model that uses pre-treatment CT scan peritumoral radiomic features and clinical descriptors to accurately forecast pembrolizumab response. This model is a novel interpretable tool that leverages imaging features and equips clinicians to tailor first-line immunotherapy treatments, potentially improving the outcomes of patients with advanced lung cancer.

## 1. Introduction

Lung cancer (LC) is the leading cause of cancer death in Canada and worldwide with its 5-year survival among the lowest at 22% [1,2]. This is largely due to most diagnoses being made at incurable late stages, compared to over 80% five-year survival if treated in stage IA [3]. Non-small-cell lung cancers (NSCLCs) are the most common type. The first-line treatment decisions of advanced NSCLC are reliant on genetic aberrations [4], while there remains a large proportion of patients with no targetable mutations [5]. Clinical trials have shown that immune checkpoint inhibitors (ICIs), either as a single agent or a combination of immunotherapy (IO) and chemotherapy are treatment options for the patients without a targetable mutation [6,7,8,9]. After the KN-024 trial, Health Canada approved pembrolizumab monotherapy in the first-line setting for a special cohort of advanced NSCLC patients with high PD-L1 expression (i.e., ≥50%), no epidermal growth factor receptor (EGFR), or anaplastic lymphoma kinase (ALK) aberration [4]. In addition, KN-024’s 5-year survival data show that the long-term survival was mainly driven by the responders.

Despite its benefit, the trial and clinical practice also suggested that 55% of this high PD-L1 expression cohort do not respond to this simple single-agent pembrolizumab regimen, hence, they likely require alternative therapy to achieve timely disease control. Therefore, identifying non-responders prior to initiating the pembrolizumab is a critical task as this would guide first-line treatment decisions in this PD-L1-high cohort. Currently, there are no consensus biomarkers or clinicopathological features that we can employ to selecting the 55% of patients that would benefit from alternative first-line treatment.

Radiomics is an emerging field of quantitative imaging. In contrast to traditional visual interpretation, radiomics converts digital images into high-dimensional data to represent features that are imperceptible to the human eye [10,11,12]. Radiomic features can be combined with other patient data and mined with sophisticated bioinformatics tools to develop models for diagnosis, prognosis, and biological endpoint prediction [12,13,14,15,16,17,18]. This combination of radiomic and other patient features is commonly referred to as a “radiomic signature”. In lung cancer, radiomics has been shown to predict pathological response, metastasis, and survival, and to correlate with histology, mutations [19,20,21,22,23,24,25], PD-L1 expression [13,26], and immunotherapy response [17,27,28]. Trebeschi et al. found that lesion-based CT radiomics could identify ICI treatment-responding metastatic NSCLCs [21]. Sun et al. reported that a combination of radiomic signatures and clinicopathological features could predict the PD-L1 expression of NSCLC tumors [26]. Yolchuyeva et al.’s recent study showed that pre-treatment CT radiomic features could predict both PD-L1 expression and progression-free survival in a Canadian cohort of NSCLC patients [13].

Recent studies that have investigated radiomic features drawn from the peritumoral region—the area surrounding a lesion of interest—have shown these features to be promising predictors in several NSCLC-related prediction tasks [29,30,31,32,33]. These features have gained momentum as potential biomarkers, as they potentially represent the immune microenvironment surrounding the tumor, and immunotherapy exerts its anti-tumor effects by influencing both the tumor and its surrounding immune microenvironment [34]. Some studies have shown that some peritumoral features can correlate to inflammatory cells and microvessels, which are known mechanisms of response outcomes [35,36,37,38,39]. The need for additional validating studies investigating the potential of peritumoral radiomic feature predictors has been called for [29]. Therefore, it is crucial to investigate the peritumoral microenvironment’s features when investigating response to immunotherapy.

In our previous study, we demonstrated that clinical characteristics and baseline disease burden, as assessed by conventional CT findings, were significantly associated with treatment response to pembrolizumab monotherapy [40]. Building on this foundation, this pilot study aims to develop and validate a pragmatic machine learning (ML) model by integrating intra- and peritumoral radiomic features. We hypothesize that these radiomic features can enhance and complement the standard CT evaluations and clinical characteristics, improving our prior clinical model’s predictions for response to pembrolizumab in a high PD-L1 NSCLC patient cohort.

Since CT-based radiomics have high potential for clinical implementation, this pilot work represents a step toward prospectively evaluating the medical utility of in-house radiomics-based ML models in leveraging routine clinical CTs for personalized treatment planning in daily Canadian clinical practice.

## 2. Materials and Methods

### 2.1. Patients Selection Criteria

**Figure 1:**
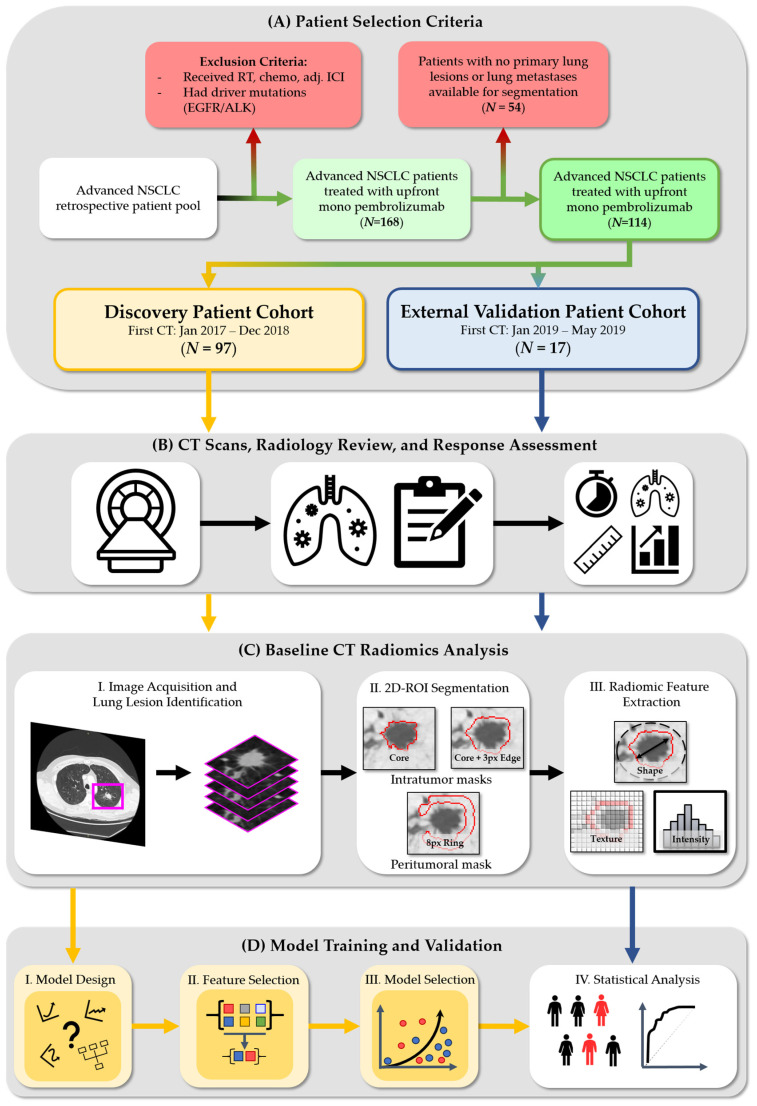
Radiomics extraction pipeline and experimental procedure. The workflow consists of the following steps: (**A**) Patient selection criteria—we retrospectively identified two patient cohorts and separated them into a discovery and external validation datasets; (**B**) CT scans, radiology review, and response assessment—CT scans from these two cohorts are assessed by two expert radiologists to determine treatment response using RECIST v.1.1; (**C**) Baseline CT radiomics analysis—scan volumes are then preprocessed by in-house segmentation pipeline that provides intra- and peritumoral segmentation masks in order to calculate shape, texture, and intensity radiomic features. (**D**) Model training and validation—the trends in the discovery dataset are used to determine the primary model type and identify the combination of features that can predict immunotherapy response. A finalized model was then selected and validated on the external set. NSCLC—Non-small-cell lung cancer, RECIST—Response Evaluation Criteria in Solid Tumors; CT—computed tomography.

A retrospective dataset from British Columbia Cancer was identified that satisfied the following eligibility criteria: males and females from all ethnic groups and racial backgrounds that received first-line single-agent pembrolizumab for stage IIIB or IV NSCLC (without EGFR/ALK aberrations), and PD-L1 expression scores ≥ 50% (determined by Standard-of-Care IHC assay at the same institution), referred between January 2017 and 31 May 2019. Patients were excluded if the following applied: they received concurrent chemotherapy; additional or adjuvant ICIs/immunotherapy; received radiation and could not evaluate treatment response to IO; had a known or suspected autoimmune disease, or received long-term immunosuppressive therapy. Patients had a baseline CT ≤ 6 weeks prior to initiating pembrolizumab and the first follow-up (FU) CT 9 to 12 weeks after treatment started. These patients were separated into two datasets: a discovery training set (Tr-set; *n* = 97, 56 F:41 M, 73 ± 6 yo, referred between January 2017 and December 2018) and an external test set (X-set; *n* = 17, 9 F:8 M, 65 ± 10 yo, referred after January 2019), as shown in Figure 1. Baseline patient demographics and clinical findings, such as age, sex, Eastern Cooperative Oncology Group (ECOG) performance status [41], smoking status, and pack-years, were collected and have been shown as discriminating features in our prior work [40] (Table 1). 

### 2.2. CT Scans, Radiology Review, and Response Assessment

The baseline and first FU CT scans were acquired according to the standard body scanning protocols at our institution as previously published [40]. All CT scans used a GE Light Speed CT scanner (GE Healthcare, Milwaukee, WI, USA). Two board-certified qualified radiologists reviewed all imaging and any disagreement between endpoint assessments was resolved by a consensus meeting while blinded to the patient outcome data to assess treatment.

Baseline CT scan assessment included the measurement of the dominant lung tumor, and the total number of organs/sites with metastasis, which was used to estimate the total disease burden. Additional diagnostic modalities (e.g., PET, MRI, US, and biopsied tissue results) were used when available to determine the distribution of metastatic disease.

The baseline and first FU CT were compared to assess the treatment response as per the Response Evaluation Criteria in Solid Tumors criteria (RECIST v1.1) as we previously described [40,42]. Progressive disease (PD) was defined when the sum diameters of all measured lesions show no less than 20% increase. Complete Response (CR) refers to the disappearance of all target lesions; and Partial Response (PR) means at least a 30% decrease in the sum diameters of target lesions. Stable Disease (SD) consisted of insufficient growth or shrinking to qualify for PD or PR. Patients in each set were categorized into two groups: PD (Tr-set: *N* = 27, *n* = 31 lesions; X-set: *N* = 9, *n* = 9 lesions), or “disease control” (DC) if they demonstrated CR, PR, or SD (Tr-set: *N* = 70, *n* = 88 lesions; X-set: *N* = 8, *n* = 10 lesions). We have grouped patients in this way as our primary endpoint is the treatment response (i.e., PD vs. DC, or non-responders vs. responders), and in this study we aim to identify PD patients that could be given alternative first-line regimens. There are a total of 21 patients across both sets that have more than one target lung lesion suitable for radiomics analysis.

### 2.3. Baseline CT Radiomics Analysis

#### 2.3.1. Image Acquisition and Lung Lesion Identification

All discrete lung lesions on the baseline CT were annotated by the same reviewing radiologist using soft tissue windows. Then, an in-house Otsu-based segmentation pipeline was developed to segment the entire lung parenchyma, lung tumors from CT volumes [43]. We first re-windowed and normalized the whole lung CT volumes to minimize any technical differences between images acquired with different scanners and/or scanning parameters. For each lesion, we utilized up to five axial slices to provide pseudo-volumetric data for feature extraction. This includes the central axial slice, also known as. the slice with the largest tumor area as identified by our radiologist team, and up to four adjacent axial view slices (two cranial steps and two caudal steps from the axial slice with the largest lesion area).

#### 2.3.2. D-ROI Segmentation

Three discrete masks were semi-automatically generated from each axial image: “Core”—the lesion core, “Core Plus Edge”—the lesion core plus the edge transitionary boundary pixels into the parenchyma, and “Ring”—an approximately 6 mm, or 8 px, peripheral ring around the lesion that captures the surrounding lung tissue information, representing the peritumoral region (Figure 1). The in-house segmentation program was constructed using MATLAB (2019b, The MathWorks Inc., Natick, MA, U.S.A.) and was built specifically for lung lesion segmentation [44,45]. The discovery training set for the machine learning model included radiomic features drawn from each lesion axial CT image (*N* = 560 slices).

#### 2.3.3. Radiomic Feature Extraction

From each mask (Core, Core Plus Edge, Ring), shape, texture, and intensity-based quantitative radiomics were extracted with an in-house feature bank calculator [46]. Radiomic features generated from “Ring” can be considered a 2D representation of the established 3D “shell feature” [47].

Several steps were taken to ease the concerns of model generalizability on out-of-distribution data that may be contained in the external test set: (i) categorical variables (e.g., sex, smoking status, lung cancer staging) were one-hot encoded; (ii) each individual numerical feature in the discovery training set was rescaled to a value between −1 and 1 based on the range of values for that feature, and then the learned scaling parameters were applied to the external test set; (iii) a first pass filtering operation (Supplementary Materials) was applied to remove weak, confounding, and redundant features before any feature selection algorithm was applied to the discovery training set. Clinically determined features were excluded from this filtering operation.

### 2.4. Model Training and Validation

#### 2.4.1. Model Design

A 5-fold nested cross validation logistic regression (LR) was chosen as the multivariate foundational prediction model construction method. This primary model paradigm was chosen for several reasons: (i) the inclusion of categorical variables such as patient demographic variables are more easily handled by LR models during feature selection; (ii) it is a white-box method in which the importance of the input variables are directly assessable through their interpretable model beta coefficient values; (iii) the lower likelihood of overfitting to a modestly sized and class-imbalanced dataset (LR models have much more limited degrees of freedom during training, reducing the probability of overfitting that many other more complex methods with potentially much higher degrees of freedom have such as support vector machines, deep learning approaches, etc.). A more comprehensive rationale for choosing an LR as the primary prediction method can be found in the Supplemental Materials (Section S2). Nested cross-validation folds were selected randomly such that there was no patient or lesion cross-contamination between the training and test folds in the discovery training set (Supplementary Materials).

#### 2.4.2. Feature Selection

Several commonly used feature selection algorithms were used to identify discriminating features to be used for training an LR-based classifier of PD vs. DC. These algorithms include Sequential Forward (SF), minimum Redundancy Maximum Relevance (mRMR), and ReliefF [48,49]. Radiomic feature selection for model performance was evaluated with and without including the non-radiomic baseline features, providing a total of six possible radiomic signatures to verify predictive ability. Non-radiomic baseline features include the following: age, sex, smoking status, pack years, ECOG performance status, and the total number of metastatic sites per patient on the baseline CT, which have been previously tested [40]. The decision to use a maximum of five features is based on conventional guidance for statistical rules of thumb, namely the “one-in-five rule”, for limiting the number of features in the interest of avoiding overfitting [50]. In essence, the number of predictive features should ideally be no greater than *N*/5, where *N* is the number of minority cases in the overall dataset (e.g., for our study *N* = 27, thus 27/5 ≈ 5 possible discriminating features at maximum).

A threshold performance positive difference > 0.03 AUC for each additional feature to be included was implemented (Supplementary Materials, Figure S1). This was decided to limit the total number of features included during the radiomic signature building process, which can lead to model overfitting to the discovery training set.

#### 2.4.3. Model Selection

The foundational cross-validation LR method was applied to train a model using one of the six selected radiomic feature signatures identified by the feature selection algorithms. To identify the optimal model hyperparameters for each of the six potential LR models, a 5-fold cross validated grid search of possible hyperparameter values was conducted (Supplementary Materials, Tables S1–S4). To avoid any post hoc sequential evaluation bias, these six LR models were compared based on their Receiver Operating Curve (ROC) performance on the discovery training set only. Finally, the top performing radiomic feature signature from these six models was used to train a final LR model using all data in the discovery training set as the individual prediction risk model to calculate scores for the external test set. For comparison, a separate radiomic feature signature, one that does not include patient characteristics in the feature pool, was similarly trained on all the discovery training set cases. This separate model will also be tested on the external test set to highlight the added benefit of synergizing radiomic features with patient characteristics.

#### 2.4.4. Statistical Analysis

The prediction performance of the top-performing LR model was estimated and compared with ROC analysis, and area under the curve (AUC), sensitivity (SN), specificity (SP), and the F1 score at optimal cutoff thresholds (Youden-J) [51] were reported. An optimized model was selected based on the criteria of a patient-level maximized AUC cross-fold validation paradigm on the discovery training set only. Model performance in the external test set was determined with ROC analyses as well as reporting confidence intervals for AUC. Confidence intervals on the external test set were calculated using the Fast DeLong method [52,53].

**Figure 2:**
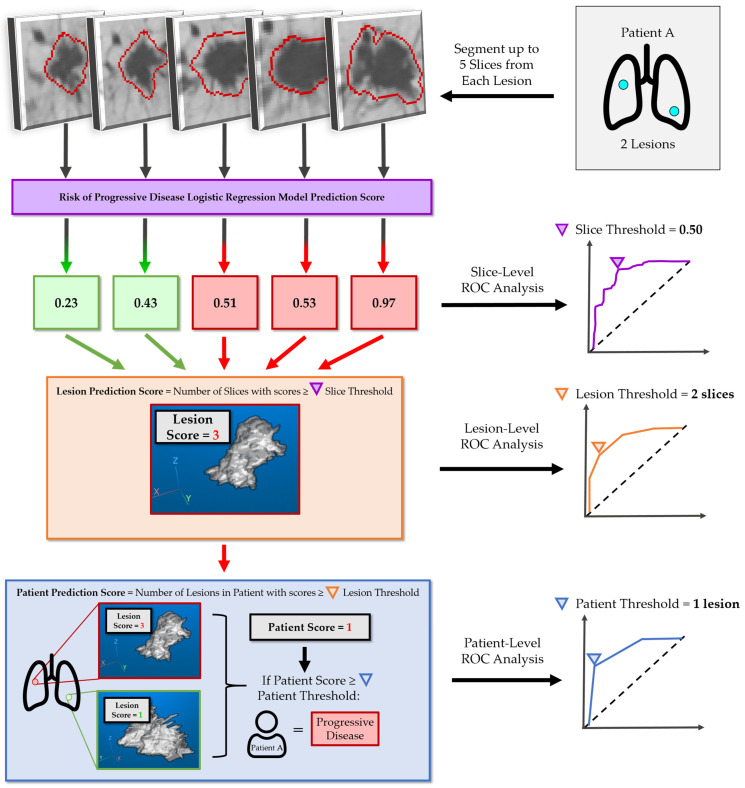
Example process detailing the frequency scoring method and slice-to-patient level prediction pipeline. All values shown here are examples and not the final scores or thresholds. First, we generate a risk of progressive disease score for each CT slice of a lung lesion. Next, using the slice-level ROC optimal threshold (e.g., X = 0.50 as shown on the example slice-level AUC), we assign a lesion-level prediction score equal to the number of slice scores ≥ X. Using the lesion-level ROC optimal threshold (e.g., Y = 2 slices), we assign a patient-level prediction score equal to the number of lesions with scores ≥ Y. Using the patient-level ROC, we then determine the optimal number of lesions that are positive to predict a patient with a likelihood of progressive disease (e.g., Z = 1 lesion). The frequency scoring method will be applied to both the discovery and external test sets. However, prediction score thresholds (i.e., X, Y, Z) at each level will be determined on the discovery training set during ROC analysis, and then those thresholds are applied to the external test set.

These six LR models were trained to evaluate if an individual axial slice from a lesion could predict progression (i.e., a CT slice-level prediction). In order to determine if a model could predict PD at the lesion level, we implemented a frequency thresholding operation that prioritized high sensitivity to granular changes across all CT slices of the lesion of interest (i.e., a lesion-level prediction). The operation counted the frequency of slices predicting PD to then generate a lesion-level classification as either PD or DC (e.g., if X/5 slices for a particular lesion were above the slice-level cutoff threshold, then the lesion had a score of X, and depending on the lesion level threshold, the lesion was classified as PD or DC). A visual example of this process is shown in Figure 2. The frequency thresholding method is intended to be a conservative approach to maintain robust lesion-level and patient-level classification of PD lesions. A lesion-level “risk of progression” prediction was generated using an optimized model trained with a combination of radiomics and patient characteristics, and it was considered to be synonymous to a patient-level risk prediction (i.e., if any lesion predicted was predicted to be PD, the patient was predicted to be PD. Both the patient- and lesion-level statistics were reported.

Patients were then separated into two risk groups: “low” or “high risk of progression” using the predicted “risk of progression” score as determined by the lesion-level LR model’s predictions. The progression-free survival (PFS; time from starting pembrolizumab treatment to the earliest CT date that showed disease progression) and overall survival (OS; time from starting pembrolizumab to death or last FU scan date) between these two groups was compared using a Mantel–Cox log-rank test [54].

All model construction and statistical analysis was performed with Python software (v3.6.5, Anaconda Inc., Austin, TX, USA) on the PyCharm platform (2019.2.2 Community Edition, JetBrains s.r.o., Prague, Czech Republic).

## 3. Results

### 3.1. Feature Selection

A number of commonly used feature selection algorithms were used to identify up-to five highly discriminating features to be used for training a logistic regression-based classifier on this PD vs. DC task. A first pass filtering operation (Supplementary Materials Section S4) removed 339 (out of 522 possible, Table S5) features. The remaining features selected for each feature selection algorithm and their individual performances are shown in Table 2. Comparisons of the six models shown in Table 2 revealed that model 5, a radiomics signature selected by SFS from a feature bank that included both radiomic features and baseline CT characteristics, was identified as the best model given its high AUC score and low cross-validation error bounds.

### 3.2. Performance of Multivariate Logisitic Regression Model Classifier

**Figure 3:**
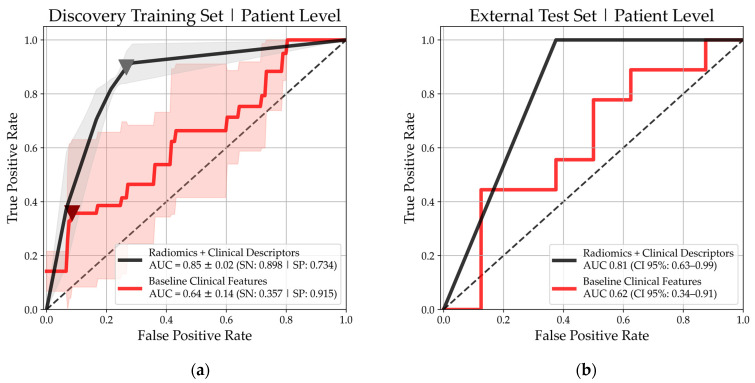
ROC analysis of the 5-fold cross validated logistic regression (LR) model (black) and a baseline CT (BCT) classification model (red) performance on the discovery and external test set when trained on a combination of radiomic and baseline CT clinical/patient descriptor features. ROCs are plotted at the patient-level using a frequency scoring method at lesion level with an optimal axial CT image slice-level prediction threshold. The Youden-J threshold index is marked on the discovery set with a triangle for both models. (**a**) LR model predictive ability on the discovery training set during 5-fold cross validation (AUC: 0.85 ± 0.02; Sensitivity: 0.898; Specificity: 0.734); (**b**) predictive ability of the discovery-trained LR model on the patient-level external test set (AUC: 0.81; CI 95%: 0.63–0.99).

As the LR model was trained to predict PD vs. DC at each 2D axial image slice of a lesion, an optimization operation was performed to find the ideal LR axial slice-level cutoff threshold that maximized the patient-level AUC on the discovery training set (Supplementary Materials). The final performance of the regression model indicated excellent and satisfactory classification ability on both the discovery and external test sets with an AUC at 0.85 ± 0.02 and 0.81 (CI 95%: 0.63–0.99), respectively (Figure 3). Operating at the Youden-J (YJ) cutoff threshold of the discovery training set, the SN for the discovery training set and external test set were 89.8% and 100.0%, respectively, while the SP was 80.0% and 62.5% (Table 3).

### 3.3. Logistic Regression Model Prediction Calculator

The model separates individual axial lesions into risk-of-progression buckets by calculating a risk score from a radiomic signature. The formula for calculating the risk score is shown in Equation (1).

(1) $$\begin{array}{ll} \mathrm{Risk}\;\mathrm{of}\;\mathrm{PD}=[1+\exp & \left(-0.282-0.509\times (\mathrm{Pack}\;\mathrm{Years}\right)\\ & +0.999\times (\mathrm{No.}\;\mathrm{of}\;\mathrm{metastatic}\;\mathrm{sites})\\ & -0.816\times (\mathrm{OD}\text{-}\mathrm{CentroidDifference}\_\mathrm{RING})\\ & +1.148\times (\mathrm{RadCentre}\_\mathrm{RING})){]}^{-1}, \end{array}$$

A visualization of SHAP values, which reflect the beta coefficients of the variables in Equation (1), are included in the Supplementary Materials (Figures S4 and S5) to provide additional context for feature importance in Equation (1).

After calculating the “Risk of PD” score for each axial slice from a lung lesion, those particular slices were then classified into the “low” or “high risk of PD” depending on if the slice’s score was above or below the YJ cutoff (YJ = 0.22) (Figure 3). Then, a risk-averse frequency thresholding method (Section 2.4.4 or Figure 2) was deployed to determine whether a lung lesion is to be classified as PD or DC. We identified that the optimal number of PD slices required for a lesion and/or a patient to be classified as PD was “2”. In other words, if two or more axial slices have a risk score above the slice-level YJ cutoff = 0.22, the lesion is classified as PD (Figures S2, S3 in the Supplemental Materials). The optimal number of lesions required to classify a patient as PD was at least “1” lesion according to the discovery training set ROC (Figure 3).

When using this frequency thresholding method, 88.9% (24/27) of PD patients in the discovery training set and 100% (9/9) of PD patients in the external test set were accurately captured in the “high risk of progression” group by the model while operating at the patient-level YJ cutoff, whereas 80.0% (56/70) and 62.5% (5/8) of DC patients in the discovery and external test set, respectively, were captured into the “low risk of progression” group as classified by the model (Table 4).

### 3.4. Survival Analysis for Predicted Low vs. High Risk of Progression Groups

**Figure 4:**
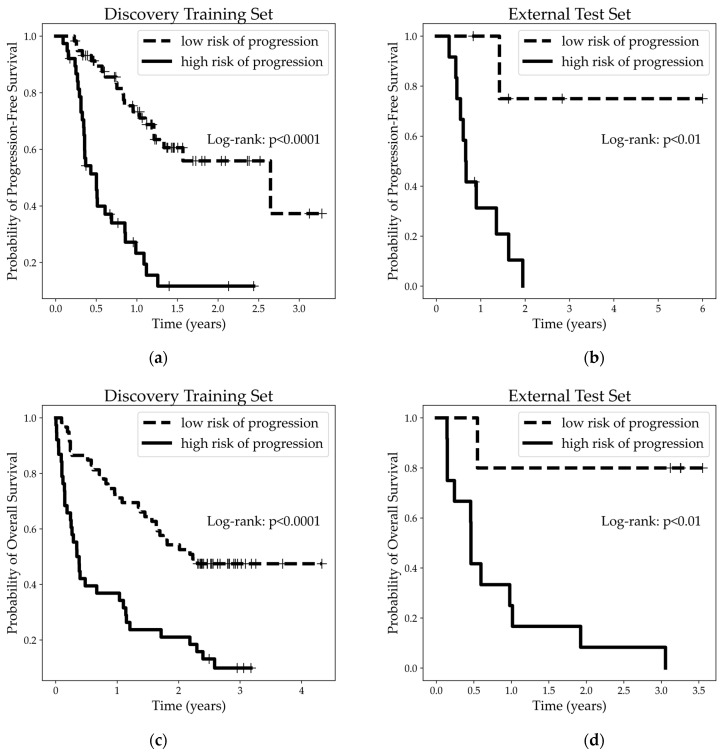
PFS and OS Kaplan–Meier plots comparing the predicted low to high risk of progression classified groups as determined by a logistic regression model for the discovery training set and external test sets of LC patients prior to treatment. Significance testing was calculated using the Mantel–Cox log-rank test. (**a**) PFS survival curves for the discovery training set (*p* < 0.0001); (**b**) PFS survival curves for the external test set (*p* < 0.01); (**c**) OS survival curves for the discovery training set (*p* < 0.0001); (**d**) OS survival curves for the external test set (*p* < 0.01).

In the discovery training set, the PFS median follow-up time was 10 months (Q1–Q3: 4.4–16; 46 survivors) and the OS median follow-up time was 16.5 months (Q1–Q3: 3.5–29.6; 32 survivors). In the external test set, the PFS median follow-up time was 10.3 months (Q1–Q3: 7.3–19.5; 5 survivors) and the OS median follow-up time was 7.1 months (Q1–Q3: 5.5–36.7; 4 survivors). With both sets of patients separated into “low” and “high risk of progression” groups as predicted by the model, Kaplan–Meier fitted survival functions were fitted to showcase the separation between the survival curves for the discovery training set (Figure 4a,c) and external test set (Figure 4b,d). The separation between the survival curves, as indicated by the significance levels of the log-rank test, shows a clear and substantial difference between the outcomes of the two predicted risk groups in both datasets for PFS and OS.

## 4. Discussion

In this pilot study, we developed an ML model integrating digital biomarkers—CT radiomic features and clinically determined values—to predict which patients would develop progressive disease (PD) to upfront pembrolizumab monotherapy vs. those with disease control (DC). The radiomic signature consists of a combination of the texture features from the peritumoral region of interest, patient smoking history, and CT estimate of tumor burden (Equation (1)). This work has advanced our prior research where only clinical and conventional CT findings were used while in a similar prediction framework [40].

In this work, we addressed how to make a binary classification—PD vs. DC, or non-responders vs. responders—in a group of otherwise almost identical NSCLC patients. We chose this binary classification because treatment switching in the clinical practice often relies on treatment response assessed radiographically at the FU CT, and it is the PD patients that we aim to identify at baseline before treatment.

From the clinical practice perspective, misclassifying PD patients as low risk of progressive disease is more detrimental, as single-agent pembrolizumab may not be optimal for non-responders that could benefit from alternative therapies for timelier disease control. In some Canadian institutions, patients cannot cross to another regimen once started on upfront pembrolizumab, emphasizing a clinical need for a high sensitivity to PD patients as the most important and desirable function of a prediction model. Our final LR model achieves a high sensitivity (Tr-set: 88.9%; X-set: 100%) which effectively identifies all PD patients, but with slightly less optimal specificity (Tr-set: 80.0%; X-set: 62.5%). Moreover, the clear separation between the response groups for both PFS and OS (Figure 4a–d) validates the model’s radiomic signature.

In this pilot study, we only performed CT radiomic feature analysis on the dominant lung lesion. We chose to do so for the following reasons: (1) To keep the prediction tool simple and therefore likely to be practical in future clinical setting. (2) A recent study compared CT radiomics from multiple organs (e.g., lung, liver, adrenal) to predict response to ICI, which showed that lung radiomics have superior performance than non-lung organs in NSCLC [21]. (3) In our model, we have already incorporated a parameter that provides the overall disease burden other than in lungs (i.e., total number of metastatic sites), hence, we believe our model reflects the overall disease burden. Having more comprehensive radiomic feature analysis of all involved organs/lesions might provide some additional information and potentially add value to the individual risk of progression. However, the complexity introduced by including all lesions from all involved organs, and in turn more confounding radiomic features across multiple lesions, could weaken the predictive power of this pilot study. The increased time and resource demands could also restrain the potential usage of this model in the clinical setting. Finally, as this work has a relatively small dataset, given the heterogeneity of the disease distribution/organ involvement among individuals (as shown in Table 1), the training regime for an organ-specific PD risk score would not likely be robust.

We implemented slice-by-slice 2D features rather than a common whole-lesion 3D feature bank for each lesion because we intended to have a predictive granularity in order to capture nuanced details throughout the lesion. For example, 3D features may only provide a single value assigned to a lesion describing say, textural density or entropy of a lesion, while the 2D version of a feature can potentially capture lesion characteristics by “scanning” slice by slice through a lesion. Therefore, 2D features can offer additional statistical insight by drawing attention to slice-by-slice texture changes throughout a lesion of interest. As there were only 18% of patients (21/114) across both datasets with more than one lung lesion available for radiomic feature analysis, that allowed us to compare the model prediction performance between the lesion level and patient levels. Our results suggest similar results between the two levels of hierarchical prediction methods (Table 4, Figure S2). This would support a potential future prediction optimization study where one uses a single dominant lung lesion to perform radiomic analysis for response prediction.

Several recent studies have found CT radiomics to be excellent predictors of varied endpoints related to immunotherapy treatment [14,15,21]. While acknowledging the significance of these studies, we are cognizant that many ML radiomics approaches do not tend to generalize well despite foundationally strong methodologies and statistical calibration efforts [28]. Moreover, each of these impressive studies tend to perhaps only answer the specific clinical question asked of their study; a clinical question that differs slightly to our own. Trebeschi et al. conducted a study where nearly all (99.2%) NSCLC patients had received previous treatment (e.g., chemotherapy, radiotherapy) and included no NSCLC patients that were treated with pembrolizumab [21]. Dercle et al. and Schroeder et al. both combined clinical characteristics and radiomic features to predict survival outcomes (OS, and PFS ≤ 90 days, respectively) which is a different prediction endpoint from this study [14,15]. Nevertheless, the tremendous works in this field have given us confidence that we are on track to provide an in-house solution to a particular clinical question in our practice: How to identify non-responders in a cohort of NSCLC patients with high PD-L1 expression prior to initiating the first-line pembrolizumab monotherapy?

Our results suggested that two discriminating peritumoral features predicted well when paired with clinical characteristics. The two geometric density metric features, the optical density centroid difference (OD-centroid difference) and the radial center (RadCentre), are statistical representations that describe the geometric density from an approximately 6 mm, or 8 pixel, extended “Ring” of tissue surrounding the lesion of interest (Figure 1). Several studies have shown peritumoral features to be good predictors of clinical outcomes in IO-treated NSCLC [31]. Li et al. found that a combination of intra- and peritumoral features could differentiate hyper-progressive disease and pseudo-progression [55]. Tunali et al. investigated the combination of peritumoral and clinical feature combinations that could predict rapid progression (i.e., time to progression (TTP) < 2 months) [56]. Wu et al. found that using a peritumoral and clinical feature nomogram, they could predict durable clinical benefit in a broad range of NSCLC (stage 1B-IV) [57]. Our findings reaffirm and support other peritumoral feature studies’ conclusions, and to our knowledge, our study is one of the few studies using CT radiomics from peritumoral features to predict the response to upfront pembrolizumab monotherapy in a Canadian cohort.

Interestingly, intratumoral radiomic signatures (i.e., features from the “Core” or “Core Plus Edge” segmentations) were not sufficiently predictive on the external test set versus the final model (Table 3). This is likely due to the strict, conservative approach we took to limit the maximum number of features to avoid model overfitting. While similarly, Huang et al. also concluded that peritumoral features made up a majority of the predictive features in their treatment response study to neoadjuvant immunotherapy [58]. Nevertheless, there remains a need for further investigation in these peritumoral radiomics features and their biological link to the treatment response [36,37,38,39].

There has been a concerted push amongst digital biomarkers researchers to ascertain a biological correlation between radiomics and established biological underpinnings of disease characteristics or pathology [19,21,59]. However, no paired immunohistochemistry slides from the described target lung lesions peritumoral region were available for this study to confirm a potential biological underpinning of these peritumoral features. Hence, for this study, we specifically and purposely have avoided drawing premature, post hoc conclusions over the exact interpretable biological nature of the lesion or parenchymal tissue characteristics that are captured by the selected peritumoral radiomic features.

Compared to the AI/ML radiomics research field, our study is not novel in terms of methodology. Rather, we prefer to think the uniqueness of our work is to explore an ML-radiomics approach in a critical unsolved real-world clinical question using a relatively new concept of including peritumoral radiomic features which can represent the composition and status of the immune microenvironment in an interpretable model. Our final goal is clinical translation, and we have made efforts to take a pragmatic approach, such as using an intuitive logistic regression model, including a small number variables in the prediction model given our modest dataset size, applying radiomics analysis to only lung lesions and incorporating the clinical features that have been previously tested valid to distinguish PD vs. DC [40]. We believe these considerations are essential to increase the medical utility of our model and facilitate research clinical translation in other Canadian institutions under the same practice guidelines and help precision oncology in a special cohort of NSCLC patients.

Our study has several limitations. First, this is a retrospective study with a small sample size and class imbalance. Given these limiting constraints, more complex models such as XGBoost, RandomForests, or Support Vector Machines (SVMs), which have shown empirically better performances, were not felt to be suitable as a primary model for this study. Second, most patients are Caucasian from a single-center treatment institution. Although this contributes to a relative homogeneity of the patient dataset characteristics, the question of generalizability and concern of racial demographics for training ML models is a commonly encountered problem for lung datasets collected in North American institutions [60]. Given the small sample size, we are unable to address the racial demographics in this study, hence this might limit its generalization in patients with different racial demographics. However, since this prediction model is intended for use in Canadian centers where the research questions arise, this racial demographic issue perhaps will not affect the potential clinical utility of the model in many Canadian settings. Another limitation arises from implementing a 2D feature bank as opposed to a more commonly used 3D feature bank. However, we believe that by applying our frequency scoring method (Figure 2), we have transitioned the prediction from individual slices represented by 2D features to whole lesion/patient classification with additional granular detail to lesion morphology, and therefore additional predictive ability.

To ensure more robust conclusions, future studies will strive to include data from multiple institutions with a diverse ethnic representation, as well as the integration of quantitative pathological features of the tumor and tumor microenvironment with the radiomic features to further enhance the accuracy of the prediction tool. Also, with more data, it becomes possible to explore additional and more complex model types, such as Random Forests or potentially deep learning architectures. The latter is an especially attractive avenue of potential research as carefully deployed fully automated workflow can further optimize the timeliness of clinical decision making.

## 5. Conclusions

Our findings demonstrate predictive associations between clinical features, pre-treatment peritumoral CT radiomics features of lung tumors, and immunotherapy response, by distinguishing responders from non-responders, in a cohort of advanced NSCLC patients that received single-agent upfront pembrolizumab.

Peritumoral radiomic features have been shown to have a biological link to the response to immunotherapy. These features require further assessment and validation as a viable method that may complement and augment conventional CT scan assessment for response prediction.

This pilot study provides a pragmatic in-house ML model that could potentially be used to predict patient response to treatment with binary classification (i.e., PD vs. DC) to facilitate the clinical treatment decision making processes in Canadian institutions under the same high PD-L1 practice guidelines. Building on the results from this pilot study and considering the wide availability of routine clinical CT scans for lung cancer patients undergoing immunotherapy, we aim to further this research by designing clinically applicable computer-assisted prediction models to elucidate medical utility, facilitate research clinical translation, and support precision oncology.

## Figures and Tables

**Table 1:** Comparison of patient characteristics between the discovery training set and external test datasets.

Baseline Patient and Lesion Descriptor	Discovery Training Set *n* = 97 Patients				External Test Set *n* = 17 Patients			
	Progressive Disease (PD) *n* = 27		Disease Control (DC) *n* = 70		Progressive Disease (PD) *n* = 9		Disease Control (DC) *n* = 8	
**Age [median, Q1–Q3]**								
	70	65–76	74	70–79	63	57–67	64	59–74
**Sex** (*n*, col%)								
Female	14	51.9	42	60	6	66.7	3	37.5
Male	13	48.1	28	40	3	33.3	5	62.5
**Smoking status** (*n*, col%)								
Light and non-smoker	2	7.4	2	2.9	0	0	1	12.5
Smoker (current/ex)	24	88.9	65	92.9	9	100	7	87.5
Unknown	1	3.7	3	4.3	0	0	0	0
**Pack years** [median, Q1–Q3]								
	33	20–49	45	27–50	32	25–40	51	32–68
**Cancer subtype** (*n*, col%)								
Non-SCC	22	81.5	58	82.9	4	44.4	6	75
SCC	5	18.5	8	11.4	5	55.6	2	25
Not specified	0	0	4	5.7	0	0	0	0
**ECOG score** [median, col%]								
≤1	13	48.1	39	55.7	4	44.4	7	87.5
>1	12	44.4	22	31.4	5	55.6	1	12.5
Unknown	2	7.4	9	12.9	0	0	0	0
**Metastatic sites** (*n*, col%)								
Lung metastasis	16	59.3	40	57.1	5	55.6	6	75
Lymph node	24	88.9	55	78.6	9	100	3	37.5
Adrenal	10	37	10	14.3	4	44.4	0	0
Liver	5	18.5	5	7.1	1	11.1	0	0
Bone	14	51.9	18	25.7	3	33.3	1	12.5
Brain	6	22.2	21	30	2	22.2	3	37.5
Other	14	51.9	30	42.9	3	33.3	0	0
**Total No. of metastatic sites per patient** [median, Q1–Q3]								
	3	2–4	3	2–3	3	3–5	2	1–3
**Primary lung lesion size in mm** [median, Q1–Q3]								
	39.9	24.5–51.5	36	24.2–49.2	47	28–50	55	25.5–84.5

**Table 2:** Performance comparison between feature selection algorithms and the features selected. Baseline characteristics includes age, sex, smoking status, pack years, and the number of metastatic sites (full list can be found in Supplementary Materials).

Feature Selection Algorithm	Including Baseline Characteristics	ROC AUC (Discovery Training Set)	Features Selected (Up to 5)
1. mRMR *	Yes	0.77 ± 0.05	No. of metastatic sites GrayLevel6_RING FFT2_RING ECOG Score
2. mRMR *	No	0.85 ± 0.10	MinAxis_RING RadVarAngle_RING
1. ReliefF	Yes	0.67 ± 0.09	Liver Lesion V-Corre1ation_CORE
2. ReliefF	No	0.70 ± 0.08	RadSD-Angle_CORE RadKurtosis-Angle_CORE
3. SFS †	Yes	0.85 ± 0.02	Pack Years No. of metastatic sites OD-CentroidDifference_RING RadCentre_RING
4. SFS †	No	0.87 ± 0.09	TotalVariance_CORE MeanRadius_RING ODEccentricity_RING RadVarianceAngle_RING RadCentre_RING

* mRMR = minimum Redundancy Maximum Relevance, † SFS = Sequential Forward Feature Selection.

**Table 3:** Performance comparison of logistic regression models when trained with baseline clinical characteristic features, radiomics, and a combination of both feature banks.

	Discovery Training Patient Set *n* = 97 Patients				External Patient Test Set *n* = 17 Patients			
Logistic Regression Feature Type	AUC	SN	SP	F1	AUC	SN	SP	F1
Baseline Clinical Characteristics	0.64 ± 0.14	0.67	0.40	0.41	0.62 (CI 95%: 0.34–0.91)	0.89	0.25	0.70
CT Radiomic Features	0.87 ± 0.09	0.82	0.80	0.79	0.63 (CI 95%: 0.42–0.84)	0.89	0.38	0.73
Bassline Clinical Characteristics + CT Radiomic Features	0.85 ± 0.02	0.90	0.80	0.74	0.81 (CI 95%: 0.63–0.99)	1.00	0.63	0.75

**Table 4:** Comparison between discovery and external test sets for the prediction task of classifying patients with “risk of progression” scores calculated by a logistic regression model.

		Discovery Training Set *		External Test Set	
		Whole Patient-Level Reporting			
-	True Response (PD vs. DC)	PD (*n =* 27)	DC (*n* = 70)	PD (*n =* 9)	DC (*n* = 8)
Predicted risk of progression	-				
High risk of progression		24	14	9	3
Low risk of progression		3	56	0	5
		SN: 0.89	SP: 0.80	SN: 1.00	SP: 0.63
		Lesion-Level Reporting			
-	True Response (PD vs. DC)	PD (*n* = 31)	DC (*n* = 88)	PD (*n =* 9)	DC (*n* = 10)
Predicted risk of progression	-				
High risk of progression		29	36	9	4
Low risk of progression		2	52	0	6
		SN: 0.94	SP: 0.59	SN: 1.00	SP: 0.60

* Discovery training set lesion and patient totals are summed from individual folds during 5-fold cross validation. SN = Sensitivity, SP = Specificity.

## Data Availability

The data generated or analyzed during this study are available from the corresponding author by request.

## Supplementary Materials

The following supporting information can be downloaded at: https://www.mdpi.com/article/10.3390/cancers17010058/s1, Figure S1: Overall area under the ROC curve (AUC) performance across five-fold cross validation during the Sequential Forward Feature (SFS) selection process on the discovery set; Figure S2: ROC analysis of the 5-fold cross validated Logistic Regression (LR) model on discovery and external test set when trained on a combination of radiomic and baseline CT clinical/patient descriptor features; Figure S3: ROC analysis of the 5-fold cross validated Logistic Regression (LR) model on the discovery and external test set when trained on a combination of radiomic and baseline CT clinical/patient descriptor features; Figure S4: A visualization of SHAP values on the Discovery set for each individual slice (*N* = 560 slices); Figure S5: A visualization of SHAP values on the External test set for each individual slice (*N* = 94 slices); Table S1: Hyperparameter grid for logistic regression; Table S2: Hyperparameter grid for mRMR; Table S3: Hyperparameter grid for ReliefF; Table S4: Hyperparameter grid for Sequential Feature Selection; Table S5: Radiomic feature list with beta coefficient associated *p*-values determined by individually fitting single logit logistic regressions to patient outcome labels (Progressive Disease vs. Disease Control). References [40,46,61,62,63,64] are cited in the Supplementary Materials.

## Nomenclature and Acronyms

ALK	Anaplastic Lymphoma Kinase
AUC	Area Under the Curve
CI	Confidence Intervals
CT	Computed Tomography
DC	Disease Control
DL	Deep Learning
ECOG	Eastern Cooperative Oncology Group
EGFR	Epidermal Growth Factor Receptor
FU	Follow Up
ICI	Immune Checkpoint Inhibitor
IHC	Immunohistochemistry
IO	Immunotherapy
LC	Lung Cancer
LR	Logistic Regression
ML	Machine Learning
MRI	Magnetic Resonance Imaging
mRMR	minimum Redundancy Maximum Relevance
NSCLC	Non-Small-Cell Lung Cancer
OD	Optical Density
OS	Overall Survival
PD	Progressive Disease
PD-L1	Programmed Death Ligand-1
PET	Positron Emission Tomography
PFS	Progression Free Survival
PR	Partial Response
RECIST	Response Evaluation Criteria in Solid Tumors
ROC	Receiver Operating Characteristic
SD	Stable Disease
SFS	Sequential Forward Selection
SN	Sensitivity
SOC	Standard of Care
SP	Specificity
Tr-set	Discovery Training Set
US	Ultrasound
X-set	External Test Set
YJ	Youden-J

## References

[1] Brenner D.R., Gillis J., Demers A.A., Ellison L.F., Billette J.-M., Zhang S.X., Liu J.L., Woods R.R., Finley C., Fitzgerald N. (2024). Projected estimates of cancer in Canada in 2024. CMAJ.

[2] Bray F., Laversanne M., Sung H., Ferlay J., Siegel R.L., Soerjomataram I., Jemal A. (2024). Global cancer statistics 2022: GLOBOCAN estimates of incidence and mortality worldwide for 36 cancers in 185 countries. CA A Cancer J. Clin..

[3] Walters S., Maringe C., Coleman M.P., Peake M.D., Butler J., Young N., Bergstrom S., Hanna L., Jakobsen E., Kolbeck K. (2013). Lung cancer survival and stage at diagnosis in Australia, Canada, Denmark, Norway, Sweden and the UK: A population-based study, 2004–2007. Thorax.

[4] Melosky B., Blais N., Cheema P., Couture C., Juergens R., Kamel-Reid S., Tsao M.S., Wheatley-Price P., Xu Z., Ionescu D.N. (2018). Standardizing biomarker testing for Canadian patients with advanced lung cancer. Curr. Oncol..

[5] McLaughlin J., Berkman J., Nana-Sinkam P. (2023). Targeted therapies in non-small cell lung cancer: Present and future. Fac. Rev..

[6] Darvin P., Toor S.M., Sasidharan Nair V., Elkord E. (2018). Immune checkpoint inhibitors: Recent progress and potential biomarkers. Exp. Mol. Med..

[7] Reck M., Rodríguez-Abreu D., Robinson A.G., Hui R., Csőszi T., Fülöp A., Gottfried M., Peled N., Tafreshi A., Cuffe S. (2016). Pembrolizumab versus Chemotherapy for PD-L1–Positive Non–Small-Cell Lung Cancer. N. Engl. J. Med..

[8] Gandhi L., Rodríguez-Abreu D., Gadgeel S., Esteban E., Felip E., Angelis F.D., Domine M., Clingan P., Hochmair M.J., Powell S.F. (2018). Pembrolizumab plus Chemotherapy in Metastatic Non–Small-Cell Lung Cancer. N. Engl. J. Med..

[9] Paz-Ares L., Luft A., Vicente D., Tafreshi A., Gümüş M., Mazières J., Hermes B., Şenler F.Ç., Csőszi T., Fülöp A. (2018). Pembrolizumab plus Chemotherapy for Squamous Non–Small-Cell Lung Cancer. N. Engl. J. Med..

[10] Gillies R.J., Kinahan P.E., Hricak H. (2016). Radiomics: Images Are More than Pictures, They Are Data. Radiology.

[11] Mayerhoefer M.E., Materka A., Langs G., Häggström I., Szczypiński P., Gibbs P., Cook G. (2020). Introduction to Radiomics. J. Nucl. Med..

[12] Hosny A., Parmar C., Quackenbush J., Schwartz L.H., Aerts H. (2018). Artificial intelligence in radiology. Nat. Rev. Cancer.

[13] Yolchuyeva S., Giacomazzi E., Tonneau M., Lamaze F., Orain M., Coulombe F., Malo J., Belkaid W., Routy B., Joubert P. (2023). Radiomics approaches to predict PD-L1 and PFS in advanced non-small cell lung patients treated with immunotherapy: A multi-institutional study. Sci. Rep..

[14] Schroeder K.E., Acharya L., Mani H., Furqan M., Sieren J.C. (2023). Radiomic biomarkers from chest computed tomography are assistive in immunotherapy response prediction for non-small cell lung cancer. Transl. Lung Cancer Res..

[15] Dercle L., Fronheiser M., Rizvi N.A., Hellmann M.D., Maier S., Hayes W., Yang H., Guo P., Fojo T., Schwartz L.H. (2023). Baseline Radiomic Signature to Estimate Overall Survival in Patients With NSCLC. J. Thorac. Oncol..

[16] Liu Z., Zhang X.Y., Shi Y.J., Wang L., Zhu H.T., Tang Z., Wang S., Li X.T., Tian J., Sun Y.S. (2017). Radiomics Analysis for Evaluation of Pathological Complete Response to Neoadjuvant Chemoradiotherapy in Locally Advanced Rectal Cancer. Clin. Cancer Res..

[17] Sun R., Limkin E.J., Vakalopoulou M., Dercle L., Champiat S., Han S.R., Verlingue L., Brandao D., Lancia A., Ammari S. (2018). A radiomics approach to assess tumour-infiltrating CD8 cells and response to anti-PD-1 or anti-PD-L1 immunotherapy: An imaging biomarker, retrospective multicohort study. Lancet Oncol..

[18] La Greca Saint-Esteven A., Vuong D., Tschanz F., van Timmeren J.E., Dal Bello R., Waller V., Pruschy M., Guckenberger M., Tanadini-Lang S. (2021). Systematic Review on the Association of Radiomics with Tumor Biological Endpoints. Cancers.

[19] Coroller T.P., Agrawal V., Narayan V., Hou Y., Grossmann P., Lee S.W., Mak R.H., Aerts H.J. (2016). Radiomic phenotype features predict pathological response in non-small cell lung cancer. Radiother. Oncol..

[20] Coroller T.P., Grossmann P., Hou Y., Rios Velazquez E., Leijenaar R.T., Hermann G., Lambin P., Haibe-Kains B., Mak R.H., Aerts H.J. (2015). CT-based radiomic signature predicts distant metastasis in lung adenocarcinoma. Radiother. Oncol..

[21] Trebeschi S., Drago S.G., Birkbak N.J., Kurilova I., Calin A.M., Delli Pizzi A., Lalezari F., Lambregts D.M.J., Rohaan M.W., Parmar C. (2019). Predicting response to cancer immunotherapy using noninvasive radiomic biomarkers. Ann. Oncol..

[22] Song J., Shi J., Dong D., Fang M., Zhong W., Wang K., Wu N., Huang Y., Liu Z., Cheng Y. (2018). A New Approach to Predict Progression-free Survival in Stage IV EGFR-mutant NSCLC Patients with EGFR-TKI Therapy. Clin. Cancer Res..

[23] Memmott R.M., Wolfe A.R., Carbone D.P., Williams T.M. (2021). Predictors of Response, Progression-Free Survival, and Overall Survival in Patients With Lung Cancer Treated With Immune Checkpoint Inhibitors. J. Thorac. Oncol..

[24] Parmar C., Leijenaar R.T.H., Grossmann P., Rios Velazquez E., Bussink J., Rietveld D., Rietbergen M.M., Haibe-Kains B., Lambin P., Aerts H.J.W.L. (2015). Radiomic feature clusters and Prognostic Signatures specific for Lung and Head & Neck cancer. Sci. Rep..

[25] Rossi G., Barabino E., Fedeli A., Ficarra G., Coco S., Russo A., Adamo V., Buemi F., Zullo L., Dono M. (2021). Radiomic Detection of EGFR Mutations in NSCLC. Cancer Res..

[26] Sun Z., Hu S., Ge Y., Wang J., Duan S., Song J., Hu C., Li Y. (2020). Radiomics study for predicting the expression of PD-L1 in non-small cell lung cancer based on CT images and clinicopathologic features. J. X-ray Sci. Technol..

[27] Zhang C., de AF Fonseca L., Shi Z., Zhu C., Dekker A., Bermejo I., Wee L. (2021). Systematic review of radiomic biomarkers for predicting immune checkpoint inhibitor treatment outcomes. Methods.

[28] Zheng J., Xu S., Wang G., Shi Y. (2024). Applications of CT-based radiomics for the prediction of immune checkpoint markers and immunotherapeutic outcomes in non-small cell lung cancer. Front. Immunol..

[29] Wu L., Lou X., Kong N., Xu M., Gao C. (2023). Can quantitative peritumoral CT radiomics features predict the prognosis of patients with non-small cell lung cancer? A systematic review. Eur. Radiol..

[30] Bera K., Velcheti V., Madabhushi A. (2018). Novel Quantitative Imaging for Predicting Response to Therapy: Techniques and Clinical Applications. Am. Soc. Clin. Oncol. Educ. Book.

[31] Khorrami M., Prasanna P., Gupta A., Patil P., Velu P.D., Thawani R., Corredor G., Alilou M., Bera K., Fu P. (2020). Changes in CT Radiomic Features Associated with Lymphocyte Distribution Predict Overall Survival and Response to Immunotherapy in Non-Small Cell Lung Cancer. Cancer Immunol. Res..

[32] Liao C.Y., Chen Y.M., Wu Y.T., Chao H.S., Chiu H.Y., Wang T.W., Chen J.R., Shiao T.H., Lu C.F. (2024). Personalized prediction of immunotherapy response in lung cancer patients using advanced radiomics and deep learning. Cancer Imaging.

[33] Khorrami M., Jain P., Bera K., Alilou M., Thawani R., Patil P., Ahmad U., Murthy S., Stephans K., Fu P. (2019). Predicting pathologic response to neoadjuvant chemoradiation in resectable stage III non-small cell lung cancer patients using computed tomography radiomic features. Lung Cancer.

[34] Zhou F., Qiao M., Zhou C. (2021). The cutting-edge progress of immune-checkpoint blockade in lung cancer. Cell Mol. Immunol..

[35] Altorki N.K., Markowitz G.J., Gao D., Port J.L., Saxena A., Stiles B., McGraw T., Mittal V. (2019). The lung microenvironment: An important regulator of tumour growth and metastasis. Nat. Rev. Cancer.

[36] Parra E.R., Behrens C., Rodriguez-Canales J., Lin H., Mino B., Blando J., Zhang J., Gibbons D.L., Heymach J.V., Sepesi B. (2016). Image Analysis-based Assessment of PD-L1 and Tumor-Associated Immune Cells Density Supports Distinct Intratumoral Microenvironment Groups in Non-small Cell Lung Carcinoma Patients. Clin. Cancer Res..

[37] Reynders K., De Ruysscher D. (2016). Tumor infiltrating lymphocytes in lung cancer: A new prognostic parameter. J. Thorac. Dis..

[38] Hendry S.A., Farnsworth R.H., Solomon B., Achen M.G., Stacker S.A., Fox S.B. (2016). The Role of the Tumor Vasculature in the Host Immune Response: Implications for Therapeutic Strategies Targeting the Tumor Microenvironment. Front. Immunol..

[39] Vaidya P., Bera K., Gupta A., Wang X., Corredor G., Fu P., Beig N., Prasanna P., Patil P., Velu P. (2020). CT derived radiomic score for predicting the added benefit of adjuvant chemotherapy following surgery in Stage I, II resectable Non-Small Cell Lung Cancer: A retrospective multi-cohort study for outcome prediction. Lancet Digit. Health.

[40] Silver A., Ho C., Ye Q., Zhang J., Janzen I., Li J., Martin M., Wu L., Wang Y., Lam S. (2023). Prediction of Disease Progression to Upfront Pembrolizumab Monotherapy in Advanced Non-Small-Cell Lung Cancer with High PD-L1 Expression Using Baseline CT Disease Quantification and Smoking Pack Years. Curr. Oncol..

[41] Azam F., Latif M.F., Farooq A., Tirmazy S.H., AlShahrani S., Bashir S., Bukhari N. (2019). Performance Status Assessment by Using ECOG (Eastern Cooperative Oncology Group) Score for Cancer Patients by Oncology Healthcare Professionals. Case Rep. Oncol..

[42] Schwartz L.H., Litière S., de Vries E., Ford R., Gwyther S., Mandrekar S., Shankar L., Bogaerts J., Chen A., Dancey J. (2016). RECIST 1.1—Update and clarification: From the RECIST committee. Eur. J. Cancer.

[43] Janzen I., Abraham R., Seyyedi S., Khattra S., Mayo J., Yuan R., Myers R., Lam S., MacAulay C. (2022). Radiomics Based Machine Learning Model for Sub-cm Lung Nodule Malignancy Diagnosis in the PanCan Screening Study. J. Thorac. Oncol..

[44] Janzen I., Abraham R., Seyyedi S., Ho C., Melosky B., Martin M., Lam S., Yuan R., Macaulay C. (2021). P57.04 Predicting Treatment Response to 1st-line Pembrolizumab in Advanced Non-Small Cell Lung Cancer (NSCLC) Patients with High PDL1 Expression. J. Thorac. Oncol..

[45] Yuan R., Janzen I., Ho C., Melosky B., Li J., Lam S., MacAulay C. (2022). P2.09-03 A Radiomics Approach Using Baseline CT Can Predict Response to 1st-Line Pembrolizumab in Advanced NSCLC with High PD-L1. J. Thorac. Oncol..

[46] MacAulay C., Keyes M., Hayes M., Lo A., Wang G., Guillaud M., Gleave M., Fazli L., Korbelik J., Collins C. (2017). Quantification of large scale DNA organization for predicting prostate cancer recurrence. Cytom. A.

[47] Hao H., Zhou Z., Li S., Maquilan G., Folkert M.R., Iyengar P., Westover K.D., Albuquerque K., Liu F., Choy H. (2018). Shell feature: A new radiomics descriptor for predicting distant failure after radiotherapy in non-small cell lung cancer and cervix cancer. Phys. Med. Biol..

[48] Hanchuan P., Fuhui L., Ding C. (2005). Feature selection based on mutual information criteria of max-dependency, max-relevance, and min-redundancy. IEEE Trans. Pattern Anal. Mach. Intell..

[49] Robnik-Šikonja M., Kononenko I. (2003). Theoretical and Empirical Analysis of ReliefF and RReliefF. Mach. Learn..

[50] Vittinghoff E., McCulloch C.E. (2006). Relaxing the Rule of Ten Events per Variable in Logistic and Cox Regression. Am. J. Epidemiol..

[51] Youden W.J. (1950). Index for rating diagnostic tests. Cancer.

[52] DeLong E.R., DeLong D.M., Clarke-Pearson D.L. (1988). Comparing the areas under two or more correlated receiver operating characteristic curves: A nonparametric approach. Biometrics.

[53] Sun X., Xu W. (2014). Fast Implementation of DeLong’s Algorithm for Comparing the Areas Under Correlated Receiver Operating Characteristic Curves. IEEE Signal Process. Lett..

[54] Mantel N. (1966). Evaluation of survival data and two new rank order statistics arising in its consideration. Cancer Chemother. Rep..

[55] Li Y., Wang P., Xu J., Shi X., Yin T., Teng F. (2024). Noninvasive radiomic biomarkers for predicting pseudoprogression and hyperprogression in patients with non-small cell lung cancer treated with immune checkpoint inhibition. OncoImmunology.

[56] Tunali I., Gray J.E., Qi J., Abdalah M., Jeong D.K., Guvenis A., Gillies R.J., Schabath M.B. (2019). Novel clinical and radiomic predictors of rapid disease progression phenotypes among lung cancer patients treated with immunotherapy: An early report. Lung Cancer.

[57] Wu S., Zhan W., Liu L., Xie D., Yao L., Yao H., Liao G., Huang L., Zhou Y., You P. (2023). Pretreatment radiomic biomarker for immunotherapy responder prediction in stage IB-IV NSCLC (LCDigital-IO Study): A multicenter retrospective study. J. Immunother. Cancer.

[58] Huang D., Lin C., Jiang Y., Xin E., Xu F., Gan Y., Xu R., Wang F., Zhang H., Lou K. (2024). Radiomics model based on intratumoral and peritumoral features for predicting major pathological response in non-small cell lung cancer receiving neoadjuvant immunochemotherapy. Front. Oncol..

[59] Tomaszewski M.R., Gillies R.J. (2021). The Biological Meaning of Radiomic Features. Radiology.

[60] Sourlos N., Wang J., Nagaraj Y., van Ooijen P., Vliegenthart R. (2022). Possible Bias in Supervised Deep Learning Algorithms for CT Lung Nodule Detection and Classification. Cancers.

[61] Doudkine A., MacAulay C., Poulin N., Palcic B. (1995). Nuclear texture measurements in image cytometry. Pathologica.

[62] Bonferroni C. (1936). Teoria statistica delle classi e calcolo delle probabilita. Pubbl. Ist. Super. Sci. Econ. Commericiali Firenze.

[63] Spearman C. (1904). The Proof and Measurement of Association between Two Things. Am. J. Psychol..

[64] Zhang Y., Ding C., Li T. (2008). Gene selection algorithm by combining reliefF and mRMR. BMC Genom..

