# Supplementary material for: Machine Learning and Computed Tomography Radiomics to Predict Disease Progression to Upfront Pembrolizumab Monotherapy in Advanced Non-Small-Cell Lung Cancer: A Pilot Study
Source: Cancers (Basel). 2024 Dec 28;17(1):58. doi: 10.3390/cancers17010058 (PMC11719007; doi:10.3390/cancers17010058)
Supplement: Supplementary file 1 [file cancers-17-00058-s001.zip › cancers-3344352-supplementary.pdf]

Machine Learning and Computed Tomography Radiomics to Predict Disease Progression to Upfront Pembrolizumab Monotherapy in Advanced Non-Small-Cell Lung Cancer: A Pilot Study

Ian Janzen, Cheryl Ho, Barbara Melosky, Qian Ye, Jessica Li, Gang Wang, Stephen Lam, Calum MacAulay and Ren Yuan

Supplemental Materials

S1. Data Preparation Steps

S1.1. Dataset Acquisition and Separation
S1.2. Image Resampling
S1.3. Segmentation and Radiomic Feature Extraction

S2. Model Selection Criteria
S3. Model Training Methods and Hyperparameters
S4. Data and Feature Set Preprocessing
S5. Feature Selection Hyperparameters
S6. Choosing a Radiomic Signature
S7. Optimizing Patient-Level Predictions
S8. Lesion-level and Slice-level Predictions
S9. Feature Impact on Predictions
S10. Device Specs and Auto-routine run-times
S11. Feature List

References

S1. Data Preparation Steps:

The following section will primarily detail the technical aspects of the dataset, how the CT data was pre-processed, and prepped for radiomic feature extraction.

S1.1. Dataset Acquisition and Separation

- All CT scans were collected on a GE Light Speed CT scanner (GE Healthcare, Milwaukee, WI, USA)
  - o Intravenous iodinated contrast injection was obtained at the portal venous phase for the chest, abdomen, and pelvis
  - o The images were reconstructed with a 2 or 2.5 mm slice thickness imaging protocol
  - o The average native resolution was 0.72mm x 0.72mm
  - o Images were resampled to a uniform pixel size of 1mm x 1mm using a spline interpolation
- The retrospective discovery cohort (*N* = 97) of patients, referred to the clinic between January 1st 2017 and December 31st 2018, and was described in prior work [1].
- A separate, more recently collected set of patients (referred to clinic after January 1st, 2019) made up the external cohort (*N* = 30).
- Eligibility for radiomics analysis for these patients required them to have a primary or metastatic lung lesion greater than 1cm in diameter.
  - o 97 of the 138 patients in the discovery cohort satisfied this constraint.
  - o 17 of the 30 patients in the external cohort satisfied this constraint.

S1.2. Image Resampling

- Each individual CT lung volume was re-windowed to typical lung window levels:
  - o Level = -600 HU
  - o Width = 1500 HU
- Then each rewindowed lung CT volume was converted from 16-bit to 8-bit values by rescaling to a pixel value range of [0-255].

S1.3. Segmentation and Radiomic Feature Extraction

- All lung lesion segmentations were performed using an Otsu-based thresholding iterative operation based on radiologist annotated 3D bounding box coordinates for each lesion of interest.
- Up to 5 axial slice representations of each lesion, centered on the radiologist's annotated central slice, were isolated for radiomic feature extraction.
- Manual annotation was performed to erase the segmentation mask when it was shown to extend into the chest wall or airways so as to not include extreme HU

values during feature extraction in the interest of representing only lesion or parenchymal tissues.
- Axial slice images were then intensity inverted as a preprocessing step
- Axial slice images and segmentation masks were then processed by a C++ based feature bank calculator designed for cytopathological images [2] to extract all radiomic features. A full list of features can be found in Section 11 of this document.

S2. Model Selection Criteria

A Logistic Regression (LR) model was selected the primary prediction method for several reasons, both technical and qualitative, listed here:

- An LR has easily interpretable beta coefficients.
- A fair method of comparison against our previous research [1] that included some of the same discriminating features (Pack Years and No. of metastatic sites).
- Is well-suited for the intake of categorical variables (i.e., Sex, Smoking Status, ECOG Score, and the presence of metastasis at specific organ site).
- The assumption of linearity in the discovery dataset.
- The removal of variables that represent multicollinearity as per the feature filtering operation exampled in **Section 4** of this document.
- A LR is unlikely to overfit on sparse-like tabular structured datasets with careful control and optimal selection of hyperparameters.
- The discovery set, is a class imbalanced dataset, and has a modest number of patient samples and thus more complicated models that potentially have known sensitivities to these dataset issues were not evaluated. As we felt that without implementing resampling techniques during training these model might not perform repeatibly and the resampling could introduce additional class-based bias into the prediction model.
- A LR model may have a lower likelihood of overfitting with our modestly sized and class imbalanced dataset due to the lower degrees of freedom.
    - Therefore, more complex models such as XGBoost, RandomForests, or Support Vector Machines (SVMs), were not felt suitable as a primary model for this study

S3. Model Training Methods and Hyperparameters

For each of the six potential LR models, a 5-fold cross validated grid search was performed to identify the optimal hyperparameters for the given combination of features selected (**Table 2**). An optimal combination of hyperparameters was considered to be the combination that resulted in the highest AUROC score on the 5-fold cross validation (CV) discovery set. Possible hyperparameters and ranges that were included in the grid search are included in the table below, as well as the optimized hyperparameters for the optimal model as described in Section 3.3 in the main text.

Table S1. Hyperparameter grid for logistic regression.

Hyperparameter	Range	Optimized Value
**Penalty Term**	[L1, L2, ElasticNet, None]	L2
**Tolerance**	[1e-5, 1e-4, 1e-4, 1e-2]	1e-4
**Solver**	[LibLinear, L-BFGS-S[1]]	L-BFGS-S
**Class Weight**	[Balanced, None]	Balanced
**Max Iterations**	[1e2, 1e3, 1e4]	1e2

[1] Limited-memory Broyden-Fletcher-Goldfarb-Shannon Solver

To avoid sequential evaluation bias, we limited ourselves to verifying the generalizability of a finalized trained model by applying it to the external test set a single time. Results can be seen in **Table 3** and **Table 4** in the main text.

S4. Data and Feature Set Preprocessing

Several steps were taken to optimize and clean the tabular dataset for experimental execution:

- Nested cross validation folds were decided randomly using a GroupKFold operation (Scikit-learn, v.0.22.2) to maintain patient-agnostic splits between the training and test folds in the discovery set.

- Any missing radiomic feature per axial image mask was imputed using a K-Nearest Neighbor regression iterative imputer (K=3). A three tiered first-pass filtering operation was performed to reduce the number of confounding and redundant predictors. Filtering consisted of:
    - Removing predictors with zero-variance as these would have no predictive value.
    - Identifying weak predictors by individually fitting each feature with a single logit logistic regression to the patient labels and removing a feature if the p-

value associated with the beta coefficient was less than the significance threshold ($\alpha = 9.04$E-5, Bonferonni corrected [3]).
- o Removing redundant features dropping pair-wise features that had a Spearman correlation coefficient greater than 0.9 in descending order of their beta coefficient associated p-value as per step 2 above [4].

S5. Feature Selection Hyperparameters

- For each of the 3 feature selection algorithms (mRMR, ReliefF, and SFS), 5-fold CV was employed to determine the optimal predictive signature from the provided feature bank. While SFS has a built-in CV methodology, the mRMR and ReliefF python packages (PymRMR v.0.1.11, ReliefF v.0.1.2) do not. Thus, to decide the final predictive signatures, the following methodology was applied:
  - o For each CV fold, fit the feature selection algorithm to the training set and generate a potential predictive signature. Then calculate the Area Under the ROC curve (AUROC) score on the validation set fold and pair these values to the selected feature names.
  - o Identify common features across all folds and finalize these to resultant radiomic signature if the feature satisfies the following constraints:
    - ▪ Was selected in at least 3 folds
    - ▪ The AUROC score for the validation fold was greater than 0.51
  - o Identify and rank potential uncommon features (appear in 2 or less folds) by their averaged validation AUROC scores. Append the highest ranked uncommon feature to the resultant radiomic signature if the max number of features has not been reached. In the event of a tied rank between uncommon features, the uncommon feature with the lower p-value in **Table S5** will be selected.
- A grid search for the optimal hyperparameters for each feature selection algorithm was implemented. The optimal values were selected based on which combination of hyperparameters produced the maximal AUROC score on the CV discovery set. The grids for each respective feature selection algorithm are listed below for the specific model type (**Table 2** of the main text):

1) mRMR:

Table S2. Hyperparameter grid for mRMR.

Hyperparameter	Range	Optimized Value for Model #1	Optimized Value for Model #2
**Scoring**	[‘MIQ’, ‘MID’]	MIQ	MIQ

2) ReliefF

Table S3. Hyperparameter grid for ReliefF.

Hyperparameter	Range	Optimized Value for Model #3	Optimized Value for Model #4
**Number Of Neighbors**	[20, 30, 40, 50, 60, 70, 80, 90, 100]	70	80

3) Sequential Feature Selection:

Table S4. Hyperparameter grid for Sequential Feature Selection.

Hyperparameter	Range	Optimized Value for Model #5	Optimized Value for Model #6
**Direction**	[‘Forward’, ’Backward’]	Forward	Forward
**Criterion Function**	[‘Logistic Regression’, ‘Linear Discriminant Analysis’]*	Logisitic Regression	Logistic Regression

* These criterion functions implemented default hyperparameters as per Scikit-learn v.0.22.2

S6. Choosing a Radiomic Signature

Selecting a highly discriminating radiomics signature that also generalizes well to a test set is a crucial step in the prediction model process. First, we identified a number of established feature selection algorithms to choose radiomic signatures as applied to the discovery training set and our results (main text, **Table 2**) indicate that Sequential Forward Selection (SFS) algorithm achieved a superior classification performance. While SFS has a slower convergence speed than mRMR and ReliefF, the SFS method allowed us to identify clear signs of potential overfitting as well as generalization potential with the discovery set while incorporating 5-fold cross validation (**Figure S1**). While mRMR and ReliefF have been shown to have success with feature-rich datasets[5], SFS convergence and feature analysis were more desirable aspects given the trade-off of the high number of features and relatively small number of samples. Second, a maximum of 5 features was decided *a priori* (See main text, section 2.4.2) as we intended to maintain generalizability against a test set while respecting the lower number of PD patients in the discovery set. While only two

radiomic signatures resulted in a classification performance above 0.85 AUC on the discovery set, we chose the radiomic signature with the smallest error bounds to be the final predictive signature (main text, model #5, **Table 2**).

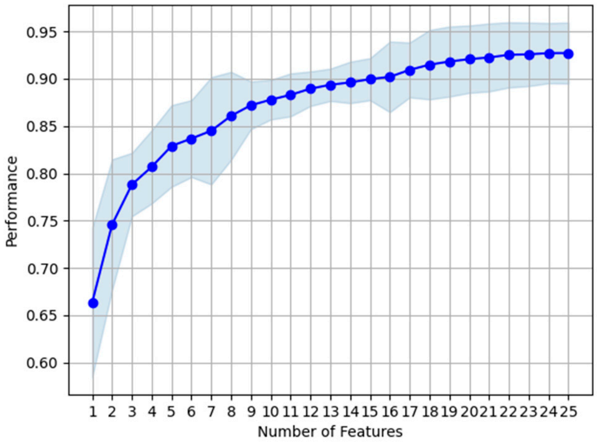

Figure S1. Overall area under the ROC curve (AUC) performance across five-fold cross validation during the Sequential Forward Feature (SFS) selection process on the discovery set. As additional features are added to the predictive signature, the overall AUC performance score increases with diminishing returns. The shaded area represents the standard deviation of the AUCs across all five-folds.

S7. Optimizing Patient-Level Predictions

To determine the optimal patient-level prediction model, we implemented a grid search of slice- and lesion-level predictive thresholds that yielded the high patient-level ROC AUC score. Using the discovery set only, a grid of possible slice prediction thresholds (i.e., fifty linearly spaced thresholds between [0.00, 1.00]) and possible lesions prediction thresholds (i.e., [0, 1, 2, 3, 4+] slices]) were iterated through during the frequency thresholding operation. The output of this grid search was the patient-level AUC score. Whichever combination of slice- and lesion-level thresholds resulted in the highest patient-level AUC score were considered to be the optimal prediction thresholds at their respective levels. In this optimization routine framework, a slice-level threshold of 0.224491, and a lesion-level threshold of 2 slices resulted in a maximized patient-level discovery set ROC AUC score of 0.85 ± 0.02.

S8. Lesion-level and Slice-level Predictions

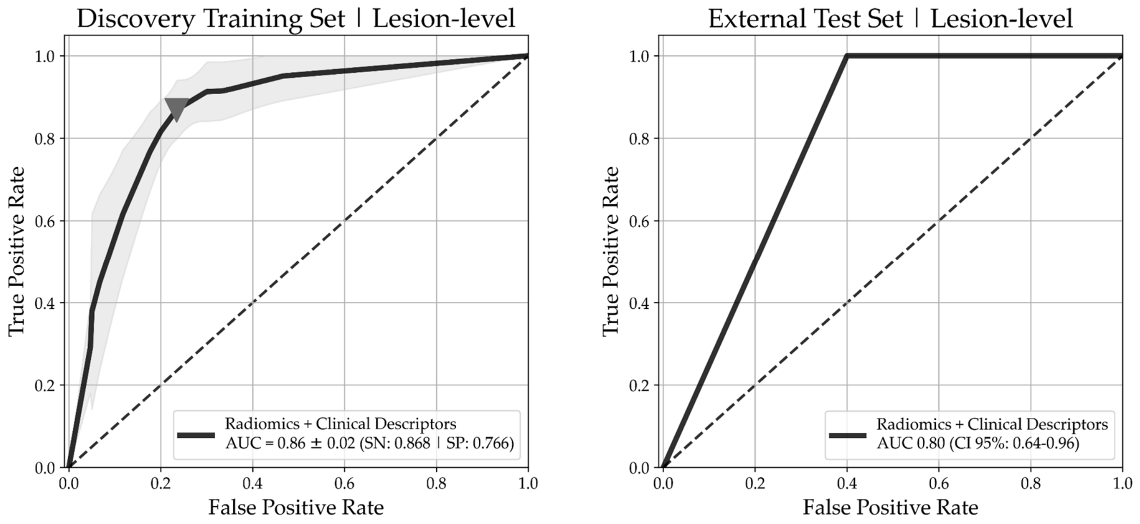

(a) (b)

Figure S2. ROC analysis of the 5-fold cross validated Logistic Regression (LR) model on discovery and external test set when trained on a combination of radiomic and baseline CT clinical/patient descriptor features. ROC Curves are plotted at the lesion-level. The Youden-J threshold index is marked on the discovery-set with a triangle for the LR model (YJ threshold = 3 slices). (**a**) LR model predictive ability on the discovery set during 5-fold cross validation (AUC: 0.87 ± 0.02; Sensitivity: 0.88; Specificity: 0.76); (**b**) predictive ability of the discovery-trained LR model on the patient-level external test set (AUC: 0.80; CI 95%: 0.64-0.96). From the discovery set, the optimal cut-off threshold is reported as 2 slices with scores greater than the optimal prediction threshold at the slice-level when implementing a frequency scoring method to the patient-level.

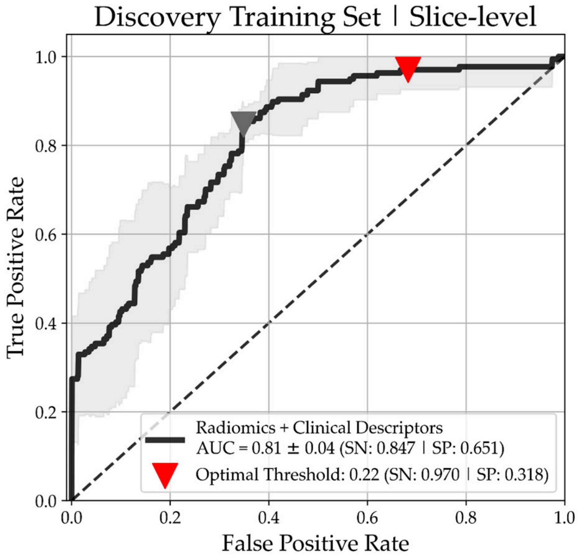

(a)

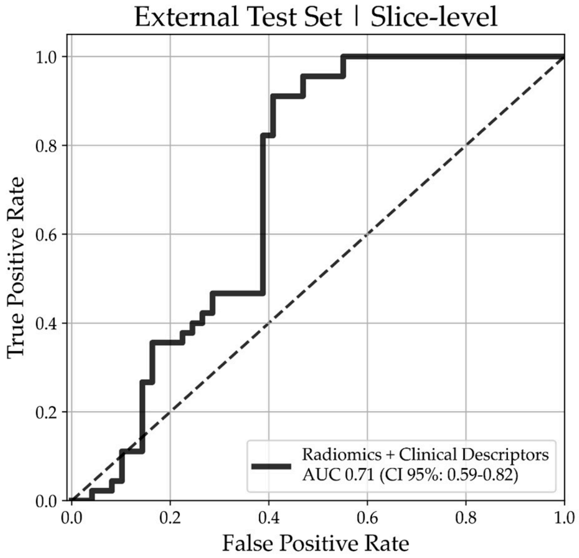

(b)

Figure S3. ROC analysis of the 5-fold cross validated Logistic Regression (LR) model on the discovery and external test set when trained on a combination of radiomic and baseline CT clinical/patient descriptor features. ROC Curves are plotted at the slice-level. The Youden-J threshold index is marked on the discovery-set with a triangle for the LR model (YJ threshold = 0.48). (a) LR model predictive ability on the discovery set during 5-fold cross validation (AUC: 0.81 ± 0.04; Sensitivity: 0.85; Specificity: 0.65); (b) predictive ability of the discovery-trained LR model on the patient-level external test set (AUC: 0.71; CI 95%: 0.59-0.82). The optimal cut-off threshold is reported as 0.22 (red marker) on the discovery set when implementing a frequency scoring method to the patient-level.

S9. Feature Impact on Predictions

Optical density centroid difference is a measure of the distance between geometric center of the mask and the density weight center of mass.
Radial centre is a difference between the geometric density centre of the object and the true centre of the boundary of the object.

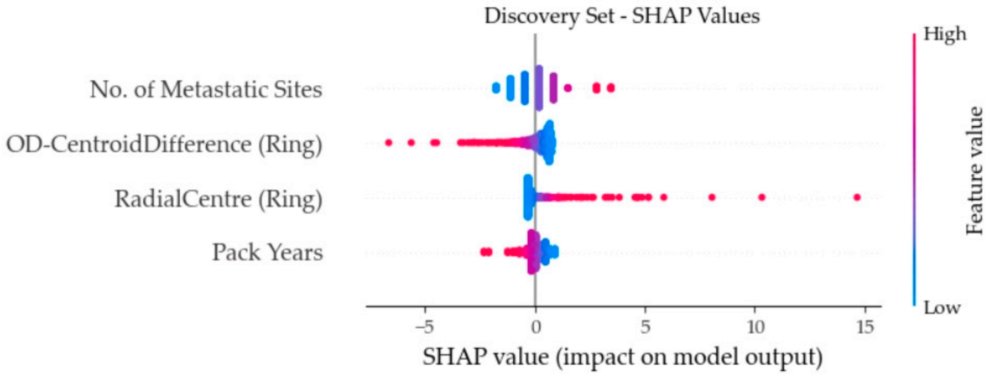

Figure S4. A visualization of SHAP values on the Discovery set for each individual slice (*N* = 560 slices). Each dot on the plot represents the contribution and feature importance of the 4 selected features in the best-performing logistic regression (LR) model. Horizontal placement of each dot indicates the magnitude of the SHAP value, and the vertical placement is captured into the feature bin. A positive SHAP value indicates a feature that positively influences the LR's prediction towards predicting the positive endpoint (Progressive Disease). The colormap (red to blue) indicates the relative feature value itself. For example, a larger Number of Metastatic Sites influences the LR model into predicting Progressive Disease, as this is reflected by more points being < SHAP value=0.

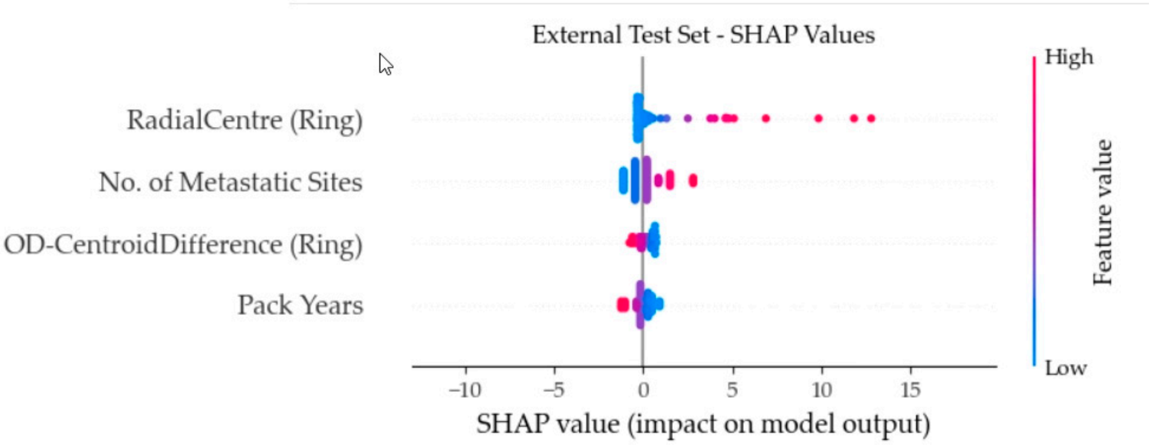

Figure S5. A visualization of SHAP values on the External test set for each individual slice (*N* = 94 slices).

S10. Device Specs and Auto-routine run-times

All experiments were performed on a Windows 10 operating system, with an AMD Ryzen Threadripper 1920X 12-Core 3.50GHz CPU, NVIDIA GeForce GTX 1050 Ti GPU, and 64GB of RAM.

All run-times are calculated from the average of 5 executions:

- Run-time for automatic lung parenchyma segmentation: 111.72s
- Run-time for automatic lung lesion segmentation: 10.26s per slice
- Run-time for Sequential Forward Feature Selection: 35.04s
- Run-time for ReliefF: 0.80s
- Run-time for mRMR: 0.53s
- Run-time for fitting LR model with 5 features: 0.11s

S11. Feature List

The following feature list was calculated using a feature bank from [2]. Additional details and applications of this cytopathological-based feature bank can also be found in [6].

The full list of possible baseline patient characteristics that feature selection algorithms were allowed to select from include:

- Age
- Sex
- ECOG Score Average
- Disease Stage (III or IV)
- Smoking Status
- Current Smoker
- Ex-Smoker
- Never Smoker
- Pack Years
- Target Lung Lesion size (mm)
- Presence of a Lung Metastasis
- Presence of Thoracic Lung Nodules
- Presence of Adrenal Metastases
- Presence of Liver Metastases
- Presence of Bone Metastases
- Presence of Brain Metastases
- Presence of Metastases in the Pleura
- Presence of Metastases in any region besides the listed above
- The Total Number of Sites with Metastases

Table S5. Radiomic feature list with beta coefficient associated p-values determined by individually fitting single logit logistic regressions to patient outcome labels (Progressive Disease vs. Disease Control).

Feature Name	Core Mask p-value*	Core Plus Edge Mask p-value*	Ring Mask p-value*
**Area**	1.527e-10	3.601e-12	7.735e-16
**AreaPRad**	1.236e-17	1.343e-19	2.647e-05
**Background †**	4.158e-26	3.197e-26	1.342e-25
**Bndvarhigh †**	2.649e-18	1.702e-23	7.958e-21
**Bndvarlow †**	1.226e-18	7.281e-20	1.917e-17
**Circularity**	1.665e-20	6.740e-24	9.654e-18
**Coverage**	1.510e-19	8.329e-23	1.465e-17
**Difforient †**	4.384e-23	3.498e-23	1.402e-21
**DNA_Amount †**	1.584e-10	9.326e-11	1.612e-01
**DNA_Cindex †**	1.584e-10	9.326e-11	1.612e-01
**DNA_Index †**	1.584e-10	9.326e-11	1.612e-01
**Eccentricity**	1.004e-22	1.453e-23	4.072e-23
**Elongation**	5.431e-20	7.792e-22	2.975e-02
**Fclusterp1 †**	2.019e-10	5.368e-08	7.620e-20
**Fclusterp2 †**	1.772e-11	6.792e-08	4.536e-20
**Fclusters1 †**	3.890e-16	9.072e-11	4.728e-01
**Fclusters2 †**	3.328e-16	8.927e-10	4.209e-02
**Fcontrast1 †**	2.842e-17	1.953e-15	2.694e-10
**Fcontrast2 †**	3.962e-16	9.282e-15	2.811e-10
**Fcorrelation1 †**	3.274e-15	3.078e-20	2.676e-10
**Fcorrelation2 †**	5.007e-13	8.752e-21	1.187e-09
**Fenergy1 †**	2.542e-21	8.499e-21	9.104e-21
**Fenergy2 †**	2.677e-20	4.697e-19	2.342e-19
**Fentropy1 †**	5.585e-25	3.121e-25	3.248e-23
**Fentropy2 †**	5.376e-25	2.458e-25	2.265e-23
**FFT10**	8.887e-17	1.490e-18	5.261e-16
**FFT11**	4.678e-18	4.524e-20	5.026e-16
**FFT12**	7.746e-16	2.106e-19	1.812e-15
**FFT2**	4.421e-16	5.550e-16	1.402e-15
**FFT3**	2.878e-16	1.877e-15	2.076e-15
**FFT4**	8.909e-15	1.666e-15	1.764e-13
**FFT5**	1.430e-16	1.048e-20	3.423e-14
**FFT6**	2.693e-15	7.372e-18	3.836e-13
**FFT7**	1.353e-17	1.183e-21	1.630e-13
**FFT8**	2.379e-17	4.767e-19	6.667e-15
**FFT9**	1.815e-17	4.996e-20	8.466e-14
**Fhomogeneity1 †**	8.687e-25	3.632e-25	1.582e-25
**Fhomogeneity2 †**	4.897e-24	5.181e-24	7.161e-25
**Fract_Area1 †**	1.521e-07	1.830e-13	6.261e-14
**Fract_Area2 †**	9.005e-09	2.520e-15	1.300e-13
**Fractal_Dim †**	5.716e-23	2.141e-24	3.742e-25
**GradNormal †**	1.013e-21	2.529e-14	8.131e-16

**Gray0_Level †**	3.477e-07	8.566e-09	1.332e-17
**Gray135_Level †**	1.316e-07	8.526e-09	1.668e-17
**Gray45_Level †**	3.024e-07	2.857e-08	4.243e-17
**Gray90_Level †**	2.710e-07	1.995e-08	7.554e-17
**Gray_Level0 †**	3.487e-07	1.590e-08	3.687e-17
**Gray_Level1 †**	1.659e-07	8.931e-09	2.931e-17
**Gray_Level2 †**	2.915e-07	4.220e-08	4.969e-17
**Gray_Level3 †**	2.276e-07	1.340e-08	2.632e-17
**Gray_Level4 †**	7.946e-03	3.108e-02	9.166e-09
**Gray_Level5 †**	2.377e-01	3.165e-01	5.033e-01
**Gray_Level6 †**	1.952e-01	3.760e-01	5.162e-01
**HighIOD †**	3.429e-02	3.390e-02	1.000
**HighArea †**	3.446e-02	3.659e-02	1.000
**HighvsLow †**	1.000	1.331e-04	1.000
**InertIODratio †**	2.836e-05	7.161e-08	1.426e-15
**Inertia †**	1.048e-23	3.394e-25	5.012e-16
**Intensity_Kurt †**	4.793e-04	2.749e-01	5.006e-08
**Intensity_SD †**	4.194e-24	1.130e-24	2.948e-17
**Intensity_Skew †**	3.613e-13	4.046e-08	2.117e-19
**IOD_Kurt †**	2.911e-24	1.976e-24	8.091e-06
**IOD_Mean †**	4.745e-22	1.573e-21	1.525e-03
**IOD_Parea †**	4.745e-22	1.573e-21	1.525e-03
**IOD_SD †**	8.972e-25	2.429e-25	1.853e-14
**IOD_Skew †**	5.305e-26	5.031e-26	6.410e-19
**LDcentdiff †**	4.014e-20	2.831e-20	1.780e-19
**LDdifforient †**	7.584e-13	4.593e-12	3.596e-07
**LDreccent †**	2.924e-24	2.601e-25	4.300e-22
**LDrinertia †**	4.229e-24	6.358e-27	2.724e-21
**LDrmaxaxis**	9.190e-25	6.277e-27	3.993e-23
**LDrminaxis**	2.061e-24	4.870e-26	4.145e-24
**Long0_Runs †**	2.845e-09	2.851e-09	1.910e-16
**Long135_Runs †**	2.017e-10	1.282e-09	3.471e-17
**Long45_Runs †**	1.000e-10	1.706e-10	1.536e-16
**Long90_Runs †**	6.091e-10	7.091e-10	9.495e-18
**Long_Run0 †**	3.051e-10	5.367e-10	2.486e-17
**Long_Run1 †**	1.187e-10	1.711e-10	2.613e-17
**Long_Run2 †**	2.074e-09	2.814e-09	1.444e-16
**Long_Run3 †**	3.004e-10	5.816e-10	1.506e-17
**Long_Run4 †**	7.706e-04	6.502e-04	1.105e-02
**Long_Run5 †**	3.188e-01	6.358e-01	4.399e-01
**Long_Run6 †**	7.579e-01	7.172e-02	1.577e-06
**LowIOD †**	5.704e-14	1.356e-19	1.171e-25
**LowArea †**	1.181e-12	7.591e-18	1.339e-25
**MaxAxis**	1.247e-18	7.656e-20	3.771e-17
**Max_Radius_M**	1.123e-18	4.249e-20	1.599e-18
**Maxradius**	1.123e-18	4.249e-20	1.599e-18
**ModehighIOD †**	1.904e-18	9.279e-18	2.145e-02
**Mean_Radius_M**	1.430e-18	4.342e-20	8.953e-16
**MeanBackground †**	4.192e-26	3.201e-26	9.706e-26
**MeanIntensity †**	2.114e-24	4.948e-25	4.294e-26

**MeanRadius**	1.430e-18	4.342e-20	8.953e-16
**MedIOD †**	1.125e-17	4.648e-17	2.145e-02
**MedArea †**	4.265e-18	2.345e-17	3.123e-02
**MedHighArea †**	3.849e-19	2.042e-18	3.123e-02
**MedHighvsLow †**	6.922e-23	1.893e-22	7.121e-01
**MedvsLow †**	3.286e-23	8.021e-23	7.121e-01
**Minaxis**	3.213e-18	6.665e-20	2.837e-13
**Min_Radius_M**	2.144e-17	1.306e-19	1.686e-05
**MinIntensity †**	3.118e-23	2.300e-23	2.259e-25
**MinRadius**	2.144e-17	1.306e-19	1.686e-05
**OD-Eccentric †**	5.604e-22	2.233e-22	5.557e-10
**OD-Inertia †**	2.496e-04	1.269e-07	3.906e-15
**OD-CentroidDifference †**	1.206e-21	2.903e-24	5.434e-25
**OD-maxAxisRatio †**	1.840e-23	2.631e-23	4.137e-10
**OD-minAxisRatio †**	4.425e-20	2.453e-23	6.008e-15
**Orientation**	2.828e-23	6.091e-23	1.227e-24
**RadCentre †**	3.184e-06	2.765e-08	1.363e-08
**RadCode †**	5.064e-25	5.409e-26	7.185e-25
**RadCvangle †**	3.807e-22	7.705e-24	7.964e-16
**RadEdge †**	7.852e-25	1.213e-24	2.511e-16
**RadEdgeVar †**	5.900e-18	8.272e-23	8.844e-21
**Radkurtangle †**	5.247e-09	4.539e-10	8.281e-01
**RadLength †**	1.852e-16	3.523e-19	1.195e-23
**RadMean †**	6.132e-23	3.884e-23	1.061e-15
**RadMeanArea †**	6.046e-06	1.371e-11	2.622e-16
**RadMinAngle †**	4.720e-26	9.890e-24	6.949e-24
**RadMinMax †**	4.738e-18	7.240e-20	7.840e-26
**RadMode †**	6.035e-18	4.784e-20	2.801e-19
**RadRangeVar †**	3.634e-17	4.150e-18	1.788e-19
**RadRatio †**	3.769e-22	1.567e-23	3.048e-11
**RadSDAngle †**	1.061e-24	1.213e-25	1.442e-23
**RadSmooth †**	2.454e-22	7.813e-25	1.483e-24
**RadVar †**	4.012e-26	5.659e-20	2.852e-19
**RadVarAngle †**	3.758e-25	6.367e-26	7.233e-27
**RadVariance †**	7.852e-09	7.425e-10	1.838e-12
**RadWeight †**	2.857e-16	4.498e-17	5.947e-13
**Run0_Length †**	5.268e-21	1.250e-22	2.759e-24
**Run0_Percent †**	4.375e-18	6.035e-18	7.041e-16
**Run135_Length †**	4.101e-21	3.036e-23	8.612e-25
**Run135_Percent †**	3.272e-17	7.693e-19	6.156e-17
**Run45_Length †**	6.945e-20	2.540e-21	8.591e-24
**Run45_Percent †**	3.905e-17	9.487e-17	1.212e-15
**Run90_Length †**	2.134e-20	6.748e-22	3.410e-24
**Run90_Percent †**	5.278e-16	6.311e-17	1.024e-15
**Run_Length0 †**	9.300e-21	3.662e-22	9.281e-24
**Run_Length1 †**	1.253e-20	1.867e-22	1.253e-24
**Run_Length2 †**	1.532e-20	1.642e-22	2.086e-24
**Run_Length3 †**	1.019e-20	2.078e-22	2.402e-24
**Run_Length4 †**	9.952e-10	9.375e-15	3.711e-24
**Run_Length5 †**	2.276e-01	8.897e-01	5.488e-01

**Run_Length6 †**	1.094e-01	4.148e-01	1.224e-11
**Run_Percent0 †**	3.579e-17	5.701e-17	4.277e-15
**Run_Percent1 †**	5.607e-17	3.799e-18	1.514e-16
**Run_Percent2 †**	1.954e-17	1.748e-17	1.466e-16
**Run_Percent3 †**	3.062e-17	6.169e-18	3.089e-16
**Run_Percent4 †**	1.377e-06	4.047e-09	1.445e-10
**Run_Percent5 †**	7.432e-01	3.725e-01	5.090e-01
**Run_Percent6 †**	1.563e-01	1.769e-01	6.728e-01
**Short0_Runs †**	4.584e-26	3.180e-25	1.312e-24
**Short135_Runs †**	6.340e-26	4.750e-26	3.043e-25
**Short45_Runs †**	1.916e-25	1.376e-24	1.186e-24
**Short90_Runs †**	1.900e-25	3.882e-25	8.299e-25
**Short_Run0 †**	9.195e-26	1.112e-25	6.955e-25
**Short_Run1 †**	8.012e-26	4.466e-25	6.450e-25
**Short_Run2 †**	3.551e-23	1.296e-23	2.204e-21
**Short_Run3 †**	8.088e-26	3.091e-25	8.127e-25
**Short_Run4 †**	3.373e-23	1.680e-21	5.069e-22
**Short_Run5 †**	1.531e-03	1.295e-06	5.677e-06
**Short_Run6 †**	5.475e-06	1.784e-12	1.007e-16
**Sphericity**	1.178e-24	8.354e-25	1.172e-08
**Stext_Orient**	6.557e-01	2.831e-23	4.883e-24
**Text_Orient**	5.064e-18	6.989e-20	6.170e-22
**Total_Variance †**	6.749e-23	9.800e-18	4.849e-10
**Vclusterp1 †**	1.256e-08	5.140e-07	5.930e-21
**Vclusterp2 †**	1.207e-09	9.384e-07	3.656e-21
**Vclusters1 †**	8.515e-13	5.489e-08	8.629e-14
**Vclusters2 †**	1.815e-12	1.090e-06	4.120e-12
**Vcontrast1 †**	4.667e-13	9.001e-18	1.616e-17
**Vcontrast2 †**	4.688e-13	4.990e-17	7.693e-20
**Vcorrelation1 †**	3.612e-19	1.833e-24	2.769e-21
**Vcorrelation2 †**	4.131e-17	8.710e-25	3.399e-19
**Venergy1 †**	2.574e-21	2.337e-20	3.660e-19

† = Texture or Intensity based feature. * = the chi-squared probability of getting a log-likelihood ratio statistic greater than the likelihood ratio chi-squared statistic

References

1. Silver, A.; Ho, C.; Ye, Q.; Zhang, J.; Janzen, I.; Li, J.; Martin, M.; Wu, L.; Wang, Y.; Lam, S.; et al. Prediction of Disease Progression to Upfront Pembrolizumab Monotherapy in Advanced Non-Small-Cell Lung Cancer with High PD-L1 Expression Using Baseline CT Disease Quantification and Smoking Pack Years. *Curr Oncol* **2023**, *30*, 5546-5559, doi:10.3390/curroncol30060419.
2. Doudkine, A.; Macaulay, C.; Poulin, N.; Palcic, B. Nuclear texture measurements in image cytometry. *Pathologica* **1995**, *87*, 286-299.
3. Bonferroni, C. Teoria statistica delle classi e calcolo delle probabilita. *Pubblicazioni del R Istituto Superiore di Scienze Economiche e Commericiali di Firenze* **1936**, *8*, 3-62.
4. Spearman, C. The Proof and Measurement of Association between Two Things. *The American Journal of Psychology* **1904**, *15*, 72-101, doi:10.2307/1412159.
5. Zhang, Y.; Ding, C.; Li, T. Gene selection algorithm by combining reliefF and mRMR. *BMC Genomics* **2008**, *9*, S27, doi:10.1186/1471-2164-9-S2-S27.

6. MacAulay, C.; Keyes, M.; Hayes, M.; Lo, A.; Wang, G.; Guillaud, M.; Gleave, M.; Fazli, L.; Korbelik, J.; Collins, C. Quantification of large scale DNA organization for predicting prostate cancer recurrence. *Cytometry Part A* **2017**, *91*, 1164-1174.
